# Transcriptional factor ATF3 promotes liver fibrosis via activating hepatic stellate cells

**DOI:** 10.1038/s41419-020-03271-6

**Published:** 2020-12-14

**Authors:** Zhemin Shi, Kun Zhang, Ting Chen, Yu Zhang, Xiaoxiao Du, Yanmian Zhao, Shuai Shao, Lina Zheng, Tao Han, Wei Hong

**Affiliations:** 1 Department of Histology and Embryology, School of Basic Medical Sciences, Tianjin Medical University, Tianjin, China; 2 Department of Hepatology and Gastroenterology, The Third Central Clinical College of Tianjin Medical University, Tianjin, China; 3 Department of Hepatology and Gastroenterology, Tianjin Third Central Hospital Affiliated to Nankai University, Tianjin, China; 4 Tianjin Key Laboratory of Artificial Cells, Artificial Cell Engineering Technology Research Center of Public Health Ministry, Tianjin, China

**Keywords:** DNA-binding proteins, Liver fibrosis

## Abstract

The excessive accumulation of extracellular matrix (ECM) is a key feature of liver fibrosis and the activated hepatic stellate cells (HSCs) are the major producer of ECM proteins. However, the precise mechanisms and target molecules that are involved in liver fibrosis remain unclear. In this study, we reported that activating transcription factor 3 (ATF3) was over-expressed in mice and human fibrotic livers, in activated HSCs and injured hepatocytes (HCs). Both in vivo and in vitro study have revealed that silencing ATF3 reduced the expression of pro-fibrotic genes and inhibited the activation of HSCs, thus alleviating the extent of liver fibrosis, indicating a potential protective role of ATF3 knockdown. However, ATF3 was not involved in either the apoptosis or proliferation of HCs. In addition, our data illustrated that increased nuclear localization of ATF3 promoted the transcription of fibrogenic genes and lnc-SCARNA10, which functioned as a novel positive regulator of TGF-β signaling in liver fibrogenesis by recruiting SMAD3 to the promoter of these genes. Interestingly, further study also demonstrated that lnc-SCARNA10 promoted the expression of ATF3 in a TGF-β/SMAD3-dependent manner, revealing a TGF-β/ATF3/lnc-SCARNA10 axis that contributed to liver fibrosis by activating HSCs. Taken together, our data provide a molecular mechanism implicating induced ATF3 in liver fibrosis, suggesting that ATF3 may represent a useful target in the development of therapeutic strategies for liver fibrosis.

## Introduction

Liver fibrosis is a consequence of almost all chronic liver diseases, which is a complication process associated with viral hepatitis B and C, alcohol abuse, nonalcoholic steatohepatitis, biliary obstruction, and several other etiologies^1–4^. It is generally accepted that hepatic stellate cells (HSCs) activation established as a central driver of fibrosis^5–8^. Fibrogenic liver cytokines including CTGF, TNF-α, and TGF-β activate HSCs and induce HSCs transformation to myofibroblasts, which synthesize large amounts of extracellular matrix (ECM) components and inhibit protease activity to decrease ECM degradation, leading to excess ECM deposition^9–11^. Moreover, activated HSCs secrete a variety of pro-fibrogenic cytokines, which aggravate the development of liver fibrosis^12^. If unresolved, the fibrotic process results in organ failure, and eventually death after the development of cirrhosis^13,14^. Therefore, a deeper understanding of the physiological and pathological mechanism of liver fibrosis is needed to develop novel strategies.

Activating transcription factor 3 (ATF3) belongs to the ATF/CREB transcription factor family that is largely diversified in size, protein sequence, and biological function. These proteins only share structurally similarity in the basic-region leucine zipper domain presumably mediating binding to a consensus DNA sequence (the ATF/CREB *cis*-element) for transcription regulation^15^. Distinct from other ATF/CREB proteins, ATF3 has a low expression level in quiescent cells, but is rapidly induced by a wide range of cellular stresses, such as injury, ischemia, or chemical toxin, as well as cytokines, such as interleukin-1β, TNF-α, and TGF-β, thus it is considered as an adaptive-response gene^16–19^. Upon activation, ATF3 translocates to the nucleus and recruits histone deacetylase1 to the promoter regions of target genes to regulate their expression^20^. Stress-inducible ATF3 can function as either a transcriptional activator or repressor, but it is still unclear whether the physiological role of ATF3 is beneficial or detrimental in the development of diseases and cell dysfunction^21–23^. Studies have shown that hepatic ATF3 protein induction promotes oxidative stress-mediated hepatic steatosis, and the development of T2D in both Zucker diabetic fatty (ZDF) rats and human subjects with nonalcoholic fatty liver disease (NAFLD)^24^. Moreover, endoplasmic reticulum (ER) stress-induced decrease of AdipoR2 that resulted from a concomitant increase in expression of ATF3, which may play a role in the development of obesity-induced insulin resistance and related ER stress in hepatocytes (HCs)^25^. On the other hand, ATF3 upregulation ameliorates ventricular remodeling and heart failure by suppressing Map2K3 expression, and subsequent p38-TGF-β signaling in cardiac fibroblasts in response to hypertensive stimuli^26^. However, the function of ATF3 has not been extensively explored in liver fibrosis. TGF-β signaling plays a central role in the initiation and progression of tissue fibrosis^27,28^. During normal wound healing, TGF-β signaling is transiently increased to activate fibroblasts^29–31^. Some studies have highlighted the important role of TGF-β/Smad3 signaling in fibrosis, and demonstrated various proteins and noncoding RNAs that were regulated by TGF-β signaling or regulated the activation TGF-β signaling during fibrogenesis. Lnc-DNM3OS was identified as a fibroblast-specific critical downstream effector of TGF-β-induced lung myofibroblast activation. While lnc-TSI specifically inhibits TGF-β-induced Smad3 phosphorylation, and downstream pro-fibrotic gene expression in the kidney and attenuated renal fibrosis^32–34^. In our previous study, it revealed that lnc-SCARNA10 functioned as a novel positive regulator of TGF-β signaling in liver fibrogenesis by promoting the expression of genes associated with ECM and TGF-β pathway^35^. However, the related regulatory elements underlying liver fibrosis require further elucidation.

In the present study, we showed that ATF3 was upregulated in carbon tetrachloride (CCl_4_) and bile duct ligation (BDL)-induced mice fibrotic livers and liver tissue samples from patients with liver fibrosis. Also, ATF3 was over-expressed in activated HSCs and injured HCs. Therefore, we explored the role of ATF3 in liver fibrosis, the data suggest that knockdown of ATF3 alleviated liver fibrosis by inhibiting the activation of HSCs in vivo and in vitro, while ATF3 was not involved in the apoptosis or proliferation of HCs. In addition, our data illustrated that ATF3 recruited SMAD3 to the promoter region and promoted the transcription of fibrogenic genes by increased nuclear location. Interestingly, ATF3 upregulated the expression of lnc-SCARNA10, which activated the TGF-β signaling in liver fibrogenesis. Furthermore, the expression of ATF3 was induced in a TGF-β/SMAD3-dependent manner, which formed a positive feedback loop. All these data suggest the TGF-β/ATF3/lnc-SCARNA10 axis contributes to liver fibrosis by activating HSCs and represents a novel therapeutic approach against liver fibrosis.

## Results

### ATF3 is over-expressed in mice and human fibrotic livers

**Fig. 1:**
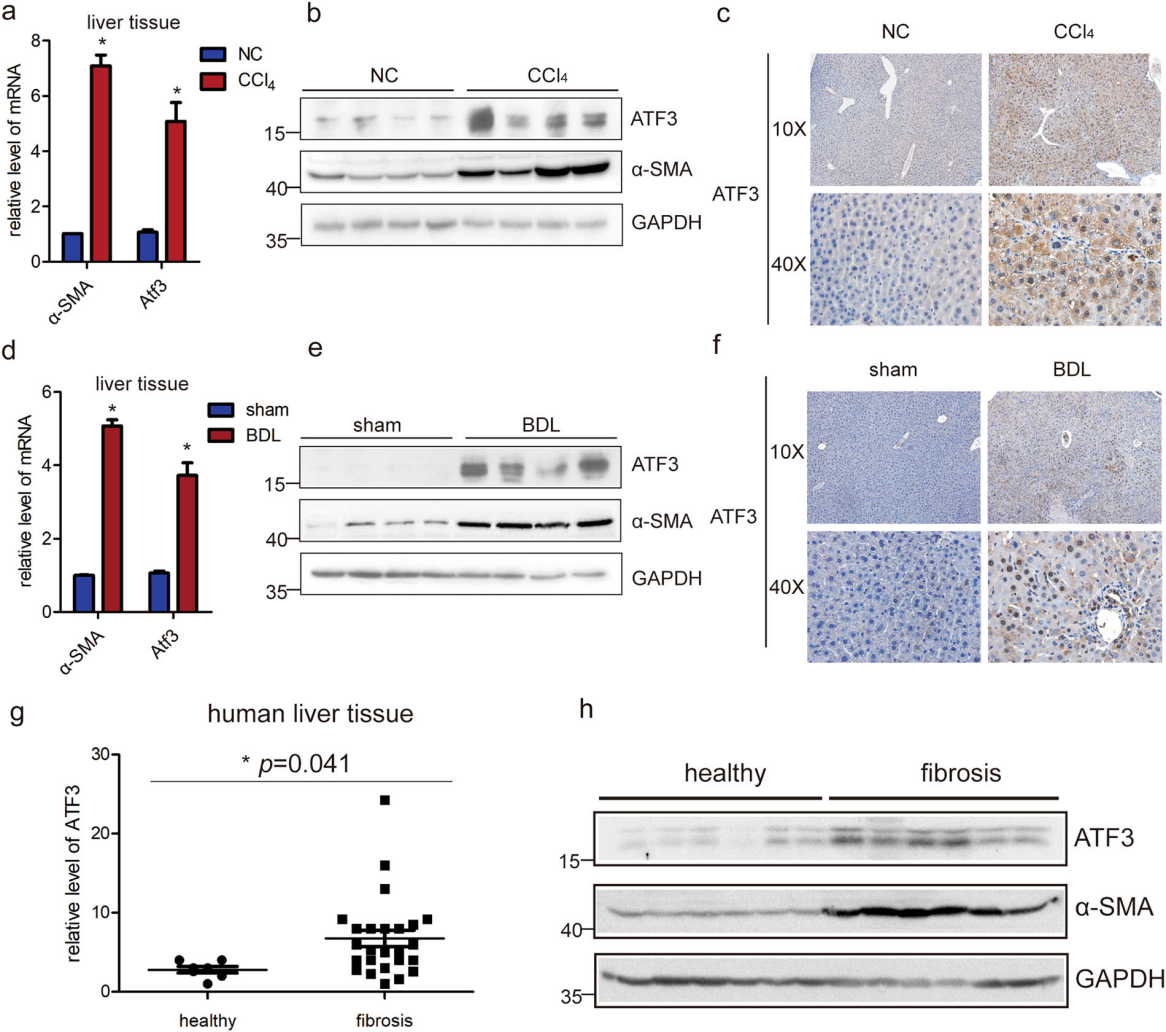
ATF3 is upregulated in mice and human fibrotic liver. **a**, **b** The expression of ATF3 and α-SMA was assessed by qRT-PCR analysis and immunoblot in livers from mice treated with CCl_4_ for 8 weeks. **c** The expression of ATF3 was examined in CCl_4_-induced fibrotic livers using IHC staining, 10× and 40× magnification. **d**, **e** The expression of ATF3 and α-SMA was assessed by qRT-PCR analysis and immunoblot in livers from mice that underwent BDL for 21 days. **f** The expression of ATF3 was examined in BDL-induced fibrotic livers using IHC staining, 10× and 40× magnification. **g**, **h** The expression of ATF3 was assessed by qRT-PCR analysis and immunoblot in liver samples of healthy people and fibrotic patients. The data were expressed as the mean ± SD for at least triplicate experiments, GAPDH was used as an internal control; *p < 0.05.

To identify the key mRNAs and lncRNAs that are potentially involved in liver fibrosis, microarray analysis was performed in mouse fibrotic and normal livers. From the study, we noted that ATF3 was remarkably upregulated in liver fibrosis according to the microarray data^36^, as shown in Supplementary Fig. 1a, b. (The microarray data discussed in the article have been deposited in NCBI Gene Expression Omnibus and are accessible through GEO Series accession number GSE80601). The results in the array analysis were confirmed with expanded samples of two mice liver fibrosis model. Consistent with the microarray data, both the mRNA level of ATF3 and α-SMA, a well-established marker of activated HSCs in the fibrotic livers, was significantly increased in CCl_4_-induced mice fibrotic liver by qRT-PCR (Fig. 1a). Furthermore, the protein level of α-SMA and ATF3 was assessed by immunoblot and IHC, the results revealed that the expression of α-SMA and ATF3 was increased (Fig. 1b, c). Also, the results were confirmed in a BDL-induced mice liver fibrosis model (Fig. 1d–f). Moreover, to explore the translational value of animal model results, we investigated whether ATF3 expression was altered in human fibrotic liver tissues. It has shown that ATF3 mRNA and protein expression were significantly increased in liver samples from patient with liver fibrosis compared to healthy liver tissues, which was consistent with the expression of α-SMA (Fig. 1g, h). Taken together, these results strongly suggest ATF3 may play a regulatory role in liver fibrosis.

### ATF3 is over-expressed in activated HSCs and injured HCs

**Fig. 2:**
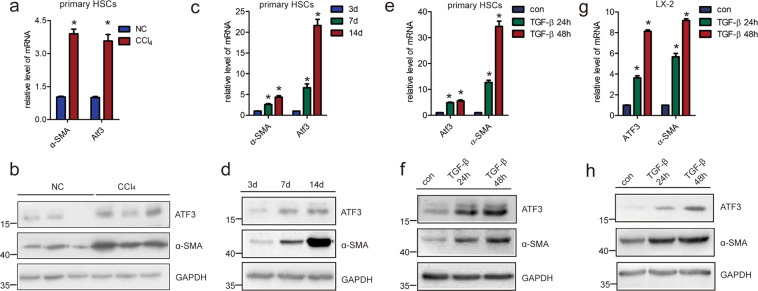
ATF3 is upregulated in activated HSCs. **a**, **b** Primary HSCs were isolated from livers of Balb/c mice treated with CCl_4_ or oil for 8 weeks, and the expression of ATF3 and α-SMA was determined by qRT-PCR and western blot. **c**, **d** The ATF3 and α-SMA level was measured by qRT-PCR and western blot in the HSCs after culture-induced activation. **e**, **f** Primary HSCs cultured at day 3 were stimulated with 10 ng/ml TGF-β for 24 or 48 h and the expression of ATF3, α-SMA was measured by qRT-PCR and western blot. **g**, **h** LX-2 cells were stimulated with TGF-β for 24 or 48 h and the expression of ATF3, α-SMA was measured by qRT-PCR and western blot. The data were expressed as the mean ± SEM for at least triplicate experiments, GAPDH was used as an internal control; *p < 0.05.

Since activated HSCs has been commonly recognized as the principal cellular players promoting synthesis and deposition of ECM proteins, we ascertained whether ATF3 was involved in HSCs activation. Upregulation of ATF3 mRNA and protein were observed in HSCs from fibrotic livers compared with the control, coinciding with an upregulation of α-SMA (Fig. 2a, b). Subsequently, qRT-PCR and immunoblot were used to assess the expression of ATF3 during the HSCs activation in vitro. The data revealed the ATF3 expression was increased in primary HSCs at day 7, and markedly increased at day 14, compared with that of day 3, which was correlated with α-SMA (Fig. 2c, d). In addition, it resulted in a significantly increased level of ATF3 and α-SMA in both mRNA and protein, when primary HSCs and human HSCs line LX-2 was stimulated with recombinant TGF-β for 24 or 48 h (Fig. 2e–h). Interestingly, the mRNA and protein level of ATF3 was increased at 2 weeks after CCl_4_ injection compared with that of 0 weeks, and gradually increased with persistent injury, and α-SMA was also gradually increased with persists CCl_4_ injection at 2, 4, 8, or 12 weeks (Supplementary Fig. 1c, d). The expression pattern of ATF3 was similar in the HSCs isolated from the liver fibrosis model in the certain time points (Supplementary Fig. 1e, f), suggesting ATF3 was an early response gene and played an important role in HSCs activation.

HCs are the dominant cells residing in the liver and HCs apoptosis has been commonly recognized as critical initiators of fibrosis in persistent liver injury^37^, thus we assessed the expression of ATF3 in injured HCs and found that the mRNA and protein level of ATF3 were over-expressed in primary HCs isolated from fibrotic livers compared with the control (Supplementary Fig. 2a, b). In addition, it resulted in a significantly increased level of ATF3 in primary HCs that as treated with TGF-β, which recognized as a potent apoptosis inducer, for 24 h (Supplementary Fig. 2c, d). Similar results were obtained in AML12 cells (Supplementary Fig. 2e, f). All these data suggest that ATF3 is upregulated in injured HCs and may involve in HCs function.

### Knockdown of ATF3 ameliorates CCl_4_-induced liver fibrosis via regulating HSCs activation in vivo

**Fig. 3:**
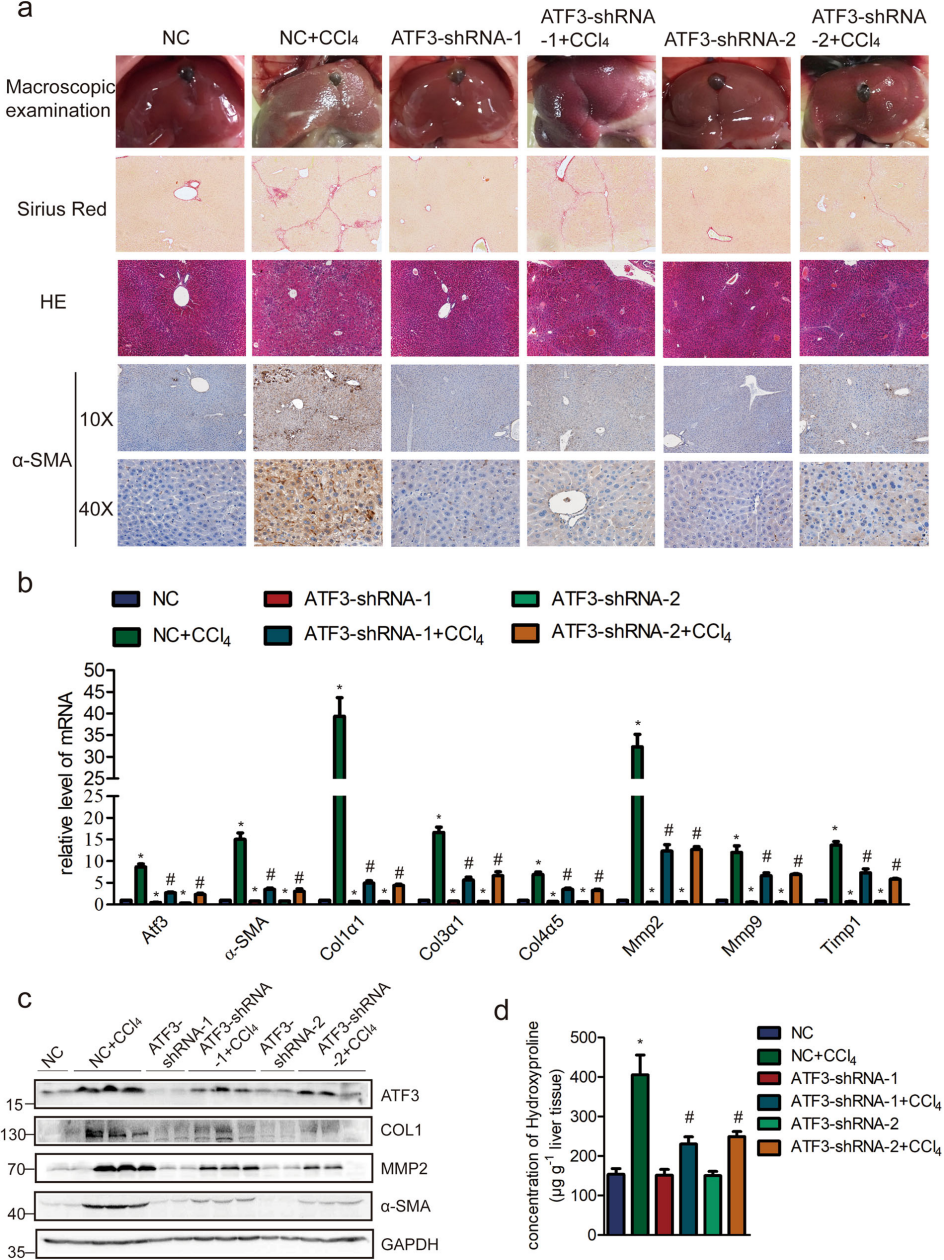
Reducing ATF3 expression ameliorates CCl4-induced liver fibrosis. Mice were treated with oil in combination with injection of lenti-NC (negative control, *n* = 10), or CCl_4_ in combination with injection of lenti-NC (NC + CCl_4_, *n* = 10), or oil in combination with injection of lenti-ATF3-shRNA1/2 (ATF3-shRNA1/2, *n* = 10), or CCl_4_ in combination with injection of lenti-ATF3-shRNA1/2, (ATF3-shRNA1/2 + CCl_4_, *n* = 10). **a** Liver fibrosis was evaluated by macroscopic examination, H&E staining, Sirius red staining, and IHC for α-SMA. **b** The mRNA level of hepatic pro-fibrogenic genes (*α-SMA, Col1α1, Col3α1,Col4α5, Mmp2*, *Mmp9*, and *Timp1)* was determined by qRT-PCR. **c** The protein level of ATF3, α-SMA, COL1, and MMP2 were determined by western blot. GAPDH was used as an internal control. **d** Quantification of hepatic hydroxyproline content. The data were expressed as hydroxyproline (μg)/liver wet weight (g). The data were expressed as the mean ± SEM for at least triplicate experiments, */#*p* < 0.05; *p < 0.05 for vs NC; #*p* < 0.05 for vs NC + CCl_4_.

Having demonstrated that ATF3 was significantly upregulated in liver fibrosis, we next investigated whether increased ATF3 contributed to liver fibrosis in vivo. ATF3 was knocked down with two separated ATF3-shRNAs, lenti-ATF3-shRNA1, lenti-ATF3-shRNA2, or lenti-NC was intravenously injected into CCl_4_-treated or oil-treated mice via the tail vein 2 weeks after the first injection of CCl_4_. After total 8 weeks of CCl_4_ treatment, whole liver extracts, primary HSCs and HCs were collected to determine the extent of liver fibrosis. The mice of CCl_4_ group infected with lenti-NC developed severe liver fibrosis, while the extent of liver fibrosis was greatly reduced in ATF3 knockdown mice as demonstrated by macroscopic examination, H&E staining, Sirius red staining, IHC for ATF3, α-SMA, and collagen1 (Fig. 3a and Supplementary Fig. 3). Furthermore, the mRNA level of ATF3 and pro-fibrogenic genes was decreased in ATF3-shRNA+CCl_4_ group compared with CCl_4_ group by qRT-PCR (Fig. 3b). Also, immunoblot for ATF3, collagen1, MMP2, and α-SMA showed that the overexpression of these proteins induced by CCl_4_ was significantly inhibited when ATF3 was knocked down (Fig. 3c). In addition, quantification of liver hydroxyproline content was also significantly decreased in CCl_4_ group mice infected lenti-ATF3-shRNAs in comparison with that infected with lenti-NC (Fig. 3d). Similarly, we demonstrated that the expression of these pro-fibrogenic genes (α-SMA, Col1α1, Col1α2, CTGF, MMP2/9, and TIMP1) exhibited a notably upregulation in the primary HSCs from CCl_4_-treated mice, while significantly decreased in that from ATF3 knockdown mice with CCl_4_ treatment (Supplementary Fig. 4a, b). Moreover, the expression of ATF3 was knocked down in primary HSCs by using two specific siRNAs targeting different sites with lenti-ATF3-shRNAs, the effects of ATF3 on the pro-fibrogenic genes was confirmed by immunoblot and qRT-PCR (Supplementary Fig. 4c, d), which was consistent with the in vivo study, indicating that ATF3 deficiency significantly decreased the ECM induction and inhibited the activation of HSCs.

To ascertain whether ATF3 silencing could affect the proliferation, apoptosis or inflammation in liver fibrosis, IHC for BAX (Supplementary Fig. 5a), qRT-PCR for *Bax*, *Mcp1*, *Bcl2*, and *Pcna* (Supplementary Fig. 5b), WB for BAX, PCNA, and cleaved Caspase3 (Supplementary Fig. 5c) were performed in the whole liver tissue. The data showed that the expression of these genes was increased in CCl_4_, however, was not significantly decreased in whole liver tissue when ATF3 was knocked down, suggesting that ATF3 may not affect proliferation, apoptosis, or inflammation in fibrotic liver. Furthermore, since ATF3 was over-expressed in injured HCs, we next explored whether ATF3 regulated the apoptosis of HCs in vivo and found that the expression of the genes, such as *Bax, Mcp1, Bcl2*, and *Pcna* did not significantly decrease in primary HCs isolated from ATF3 knockdown mice with CCl_4_ treatment compared with mice under CCl_4_ treatment (Supplementary Fig. 5d), suggesting ATF3 was not involved in the regulation of apoptosis and proliferation of primary HCs. In order to further investigate the function of ATF3 in HCs in vitro, we used lentivirus vector of ATF3-shRNA to knockdown its expression in primary HCs and AML12 cells. Consistently, the expression of the pro-inflammation gene Mcp1, the proliferation gene Pcna, and the apoptosis related genes Bax, Bcl2, and cleaved Caspase3, didn’t show significant difference in ATF3-downregulated HCs (Supplementary Figs. 6a, b and 7a, b). On the other hand, the expression of the pro-inflammation and pro-apoptosis genes was unaltered when ATF3 was over-expressed in primary HCs and AML12 cells (Supplementary Figs. 6c, d and 7c, d). All the data suggested that ATF3 may not affect apoptosis or proliferation in HCs.

Taken together, these results suggest that ATF3 depletion ameliorates the progression of liver fibrosis via inhibiting HSCs activation instead of affecting HCs function.

### ATF3 activates HSCs in vitro

**Fig. 4:**
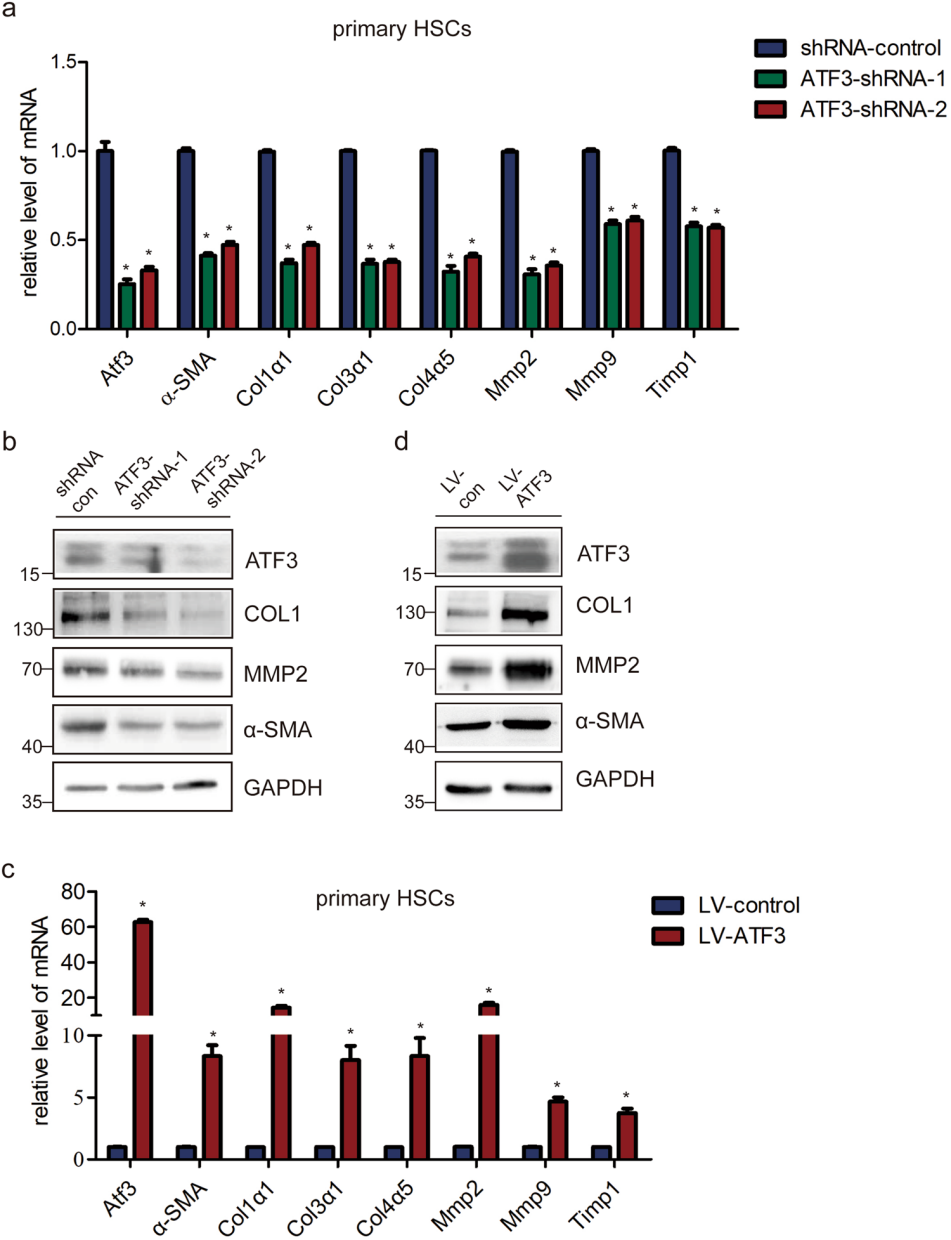
ATF3 is required for the activation of primary HSCs. **a** Primary HSCs were infected with ATF3-shRNA1/2 for 72 h. The expression of *α-SMA*, *Col1α1*, *Col3α1*, *Col4α5*, *Mmp2*, *Mmp9*, and *Timp1* was detected by qRT-PCR; *p < 0.05. **b** The protein level of α-SMA, COL1, and MMP2 were detected by western blot. GAPDH was used as an internal control. **c** The mRNA level of *α-SMA*, *Col1α1*, *Col3α1*, *Col4α5*, *Mmp2, Mmp9*, and *Timp1* were detected in primary HSCs infected with lenti-ATF3 or lenti-control by qRT-PCR; *p < 0.05. **d** The protein level of α-SMA, COL1, and MMP2 were detected in ATF3 upregulated primary HSCs by western blot. GAPDH was used as an internal control. The data were expressed as the mean ± SEM for at least triplicate experiments.

Given the evidence that ATF3 promoted the activation of HSCs in vivo, we further explored the effect of ATF3 on the activation of HSCs in vitro. The primary HSCs cells infected with ATF3-shRNAs virus showed a decreased mRNA level of the pro-fibrogenic genes *α-SMA*, *Col1α1*, *Col3α1*, *Col4α5*, *Mmp2*, *Mmp9*, and *Timp1* compared with the control (Fig. 4a). Similarly, the protein level of Col1α1, MMP2, and α-SMA was also markedly decreased by ATF3-shRNAs infection (Fig. 4b). To further investigate the role of ATF3, ATF3 was over-expressed and the data showed forced expression of ATF3 obviously increased the expression of the pro-fibrogenic genes in primary HSCs (Fig. 4c, d). Consequently, these results were further confirmed in LX-2 cells (Supplementary Fig. 8a–d), indicating that ATF3 promotes the expression of pro-fibrogenic genes and the activation of HSCs in vitro. Subsequently, in order to investigate whether ATF3 mediated the increased expression of pro-fibrotic genes induced by the TGF-β, qRT-PCR, and immunoblot were used to evaluate the mRNA and protein level of α-SMA and collagen1 in ATF3-downregulated LX-2 cells treated with or without TGF-β. The results showed that knockdown of ATF3 dramatically decreased TGF-β induced upregulation of these fibrosis-related genes in LX-2 cells (Supplementary Fig. 9a, b), suggesting that ATF3 involved in the induction of pro-fibrogenic genes by TGF-β. Taken together, our data suggest that ATF3 upregulated the expression of pro-fibrotic genes.

### ATF3 *trans*-activates the pro-fibrogenic genes

**Fig. 5:**
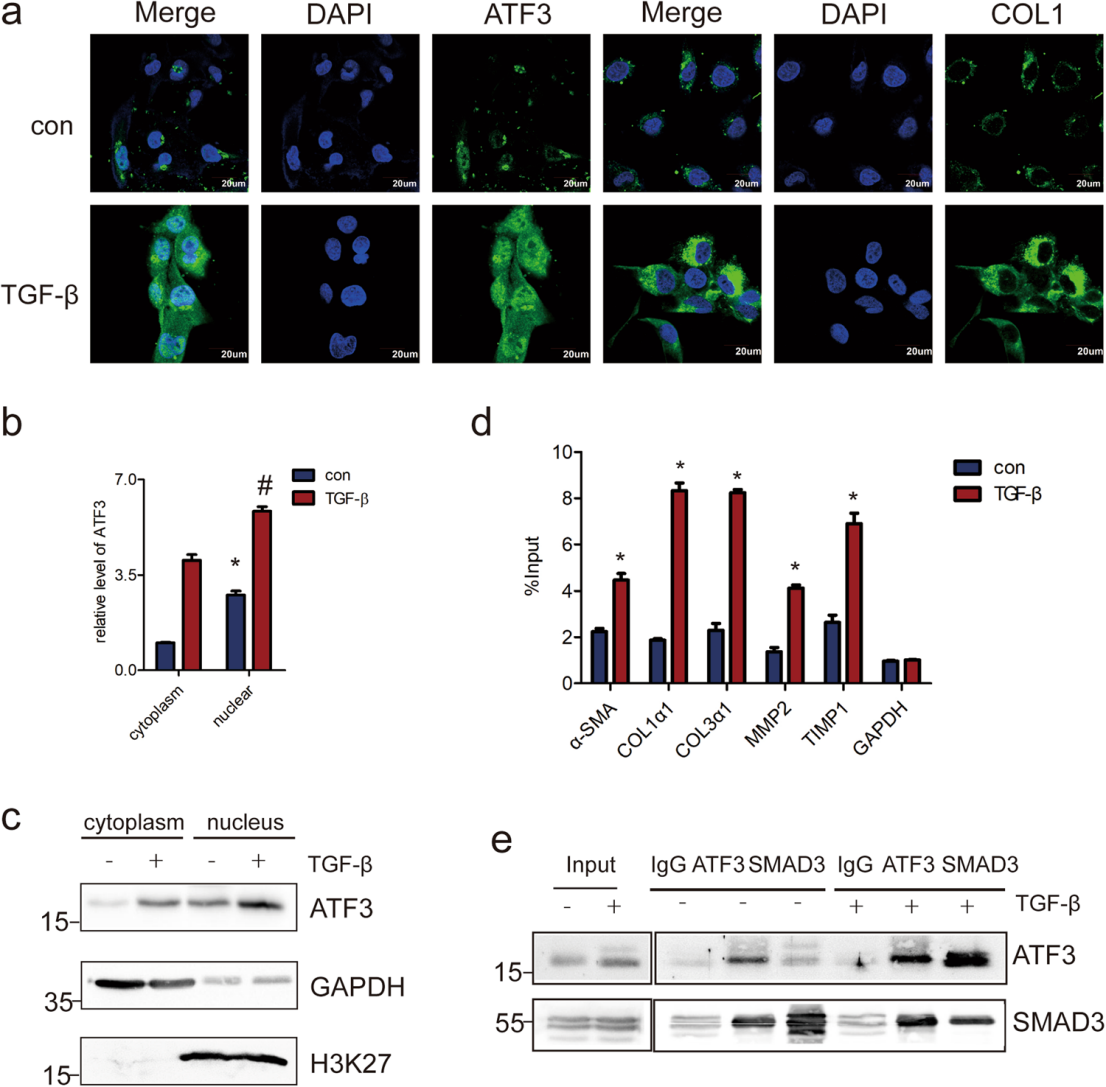
ATF3 translocates to the nucleus of LX-2 cells and promotes the transcription of pro-fibrogenic genes. **a** LX-2 cells were treated with 10 ng/ml TGF-β for 48 h, and the expression and location of ATF3 and COL1 were determined by confocal microscopy. DAPI-stained nuclei blue; scale bar, 20 μm. **b**, **c** The RNA and protein were extracted from the nuclei or cytoplasm of LX-2 cells treated with or without TGF-β, the expression of ATF3 was assessed by qRT-PCR and western blot. GAPDH was used as control in cytoplasm and H3K27 as nuclear. **d** LX-2 cells were treated with TGF-β and ChIP analyses were performed on indicated genes promoter regions, using anti-ATF3 antibody. Enrichment was shown relative to input. **e** ATF3 and SMAD3 antibodies were used for co-immunoprecipitation (IP) with LX-2 lysates treated with or without TGF-β. The data were expressed as the mean ± SEM for at least triplicate experiments. GAPDH was used as an internal control; */#*p* < 0.05.

It has been reported that upon activation, ATF3 translocates to the nucleus and regulate the transcription of target genes^20^. Therefore, we performed confocal microscopy to investigate whether the subcellular localization of ATF3 was altered in activated HSCs. When LX-2 cells were treated with TGF-β, the expression of both collagen1 and ATF3 was increased. Moreover, ATF3 was notably increased in the nucleus (Fig. 5a), indicating that ATF3 could regulate the transcription of target genes. To further confirm this finding, we investigated whether ATF3 was increased in the nucleus of primary HSCs. Primary HSCs was isolated from CCl_4_-induced liver fibrosis mice and control mice, respectively, then confocal microscopy was used to detect the expression and location of collagen1 and ATF3. The results showed that ATF3 was mostly located in the nucleus of HSCs in CCl_4_ group compared with the control group (Supplementary Fig. 10). Furthermore, when primary HSCs was activated by TGF-β treatment or culture activated at day 14, the expression of ATF3 was also increased in the nucleus (Supplementary Fig. 10a). Consistent with the finding, the mRNA and protein level of ATF3 was increased in the nucleus of LX-2 cells when treated with TGF-β by cell fractionation with qRT-PCR and western blot analysis (Fig. 5b, c). Since previous data have shown that ATF3 promotes the pro-fibrotic genes expression, we then investigated the potential mechanism by which ATF3 affected the target genes. Chromatin immunoprecipitation (ChIP) assays was performed to identify the target genes that selectively regulated by ATF3. The data showed that the enrichment of ATF3 in the promoter of pro-fibrotic genes, such as α-SMA, COL1α1, COL3α1, MMP2, and TIMP1 was significantly increased in LX-2 cells in response to TGF-β treatment (Fig. 5d), indicating that ATF3 promoted the transcription of these pro-fibrogenic genes. Moreover, it was interesting to note that co-IP assays demonstrated that SMAD3 was indeed present in the ATF3-immunoprecipitated complex (Fig. 5e). Moreover, TGF-β stimulated the recruitment of ATF3 to protein complexes containing SMAD3 (Fig. 5e), suggesting that ATF3 recruited SMAD3 to promote the expression of pro-fibrogenic genes. The results were further confirmed in primary HSCs (Supplementary Fig. 10b). Our data indicate that ATF3 mediated the upregulation of pro-fibrotic genes by TGF-β by recruiting SMAD3 to directly bind to the promoter of target genes, thus promoting the transcription of pro-fibrotic genes.

### The TGF-β/ATF3/lnc-SCARNA10 axis regulates liver fibrosis

**Fig. 6:**
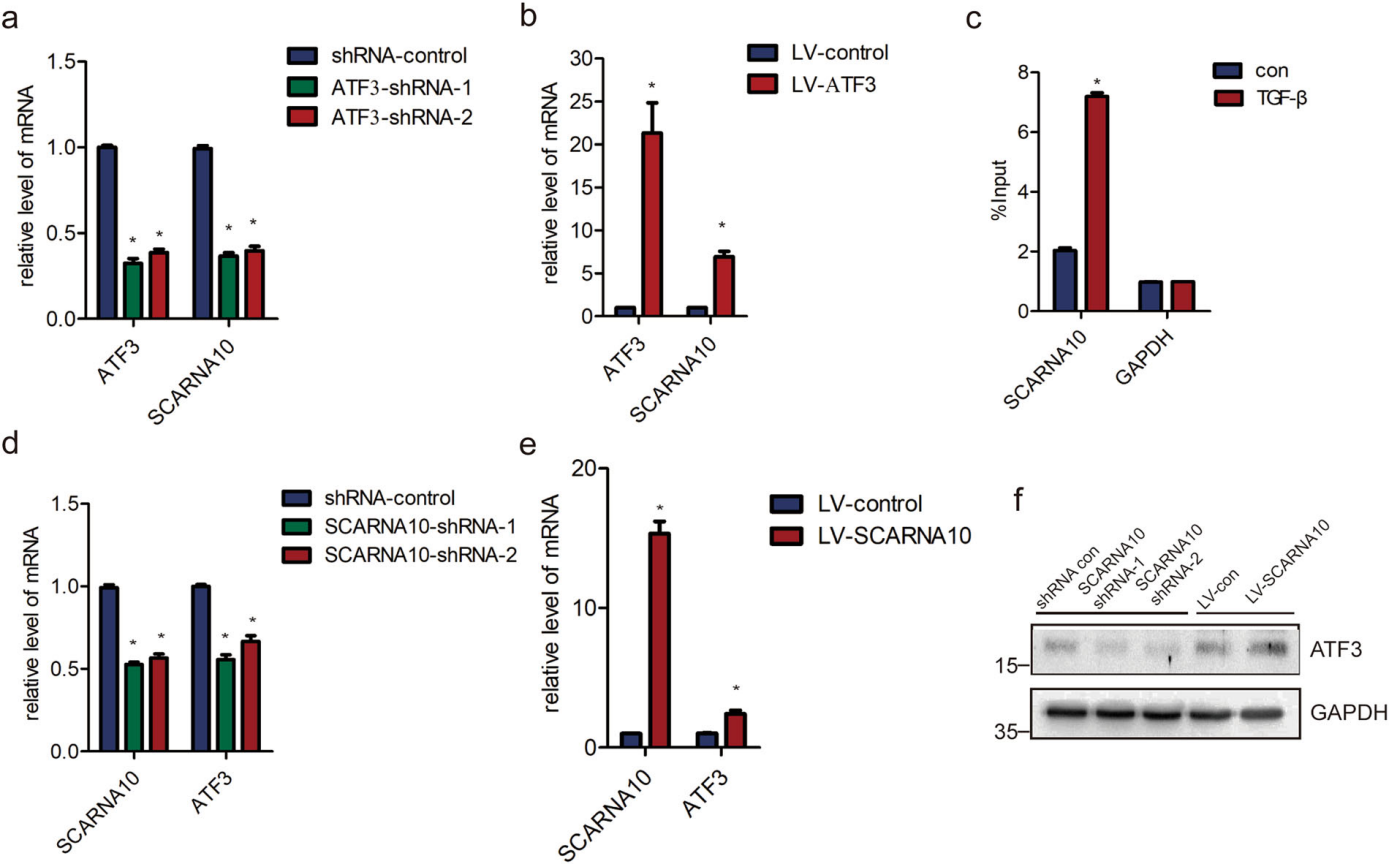
lnc-SCARNA10 forms a positive feedback loop with ATF3. **a** LX-2 cells were infected with ATF3-shRNA1/2 for 72 h. The expression of *lnc-SCARNA10* was detected by qRT-PCR. **b** The expression of *lnc-SCARNA10* was detected in LX-2 cells infected with lenti-ATF3 or lenti-control by qRT-PCR. **c** LX-2 cells were treated with TGF-β and ChIP analyses were performed on SCARNA10 promoter regions, using anti-ATF3 antibody. Enrichment was shown relative to input. **d**–**f** LX-2 cells were infected with lnc-SCARNA10 shRNA1/2 or lenti-lnc-SCARNA10 for 72 h. The expression of ATF3 was detected by qRT-PCR and western blot. GAPDH was used as an internal control. The data were expressed as the mean ± SEM for at least triplicate experiments. GAPDH was used as an internal control; *p < 0.05.

Our previous study identified a crucial role of lnc-SCARNA10, which functioned as a novel positive regulator of TGF-β signaling, promoting liver fibrosis by inducing HSCs activation^35^. It was interesting to investigate whether ATF3 and SCARNA10 could form a feedback loop with TGF-β. Promoter analysis results revealed a significant ATF3-binding promoter region of the SCARNA10 gene in JASPAR database. Moreover, SCARNA10 expression was decreased in ATF3 knockdown LX-2 cells, and increased in lenti-ATF3-transfected LX-2 cells, suggesting this transcription factor stimulates SCARNA10 expression (Fig. 6a, b). Subsequently, we explored the potential mechanism by which ATF3 promoted SCARNA10 expression. Independent ChIP assay confirmed marked enrichment of ATF3 on the promoter of SCARNA10 over normal IgG following TGF-β treatment (Fig. 6c), suggesting that ATF3 bound to the promoter of SCARNA10 and promoted its transcription. Strikingly, ATF3 mRNA and protein expression were also significantly increased when SCARNA10 was over-expressed, and decreased when SCARNA10 was knockdown in LX-2 cells (Fig. 6d–f), suggesting that ATF3 and SCARNA10 could form a positive feedback loop, which aggravated the liver fibrosis.

**Fig. 7:**
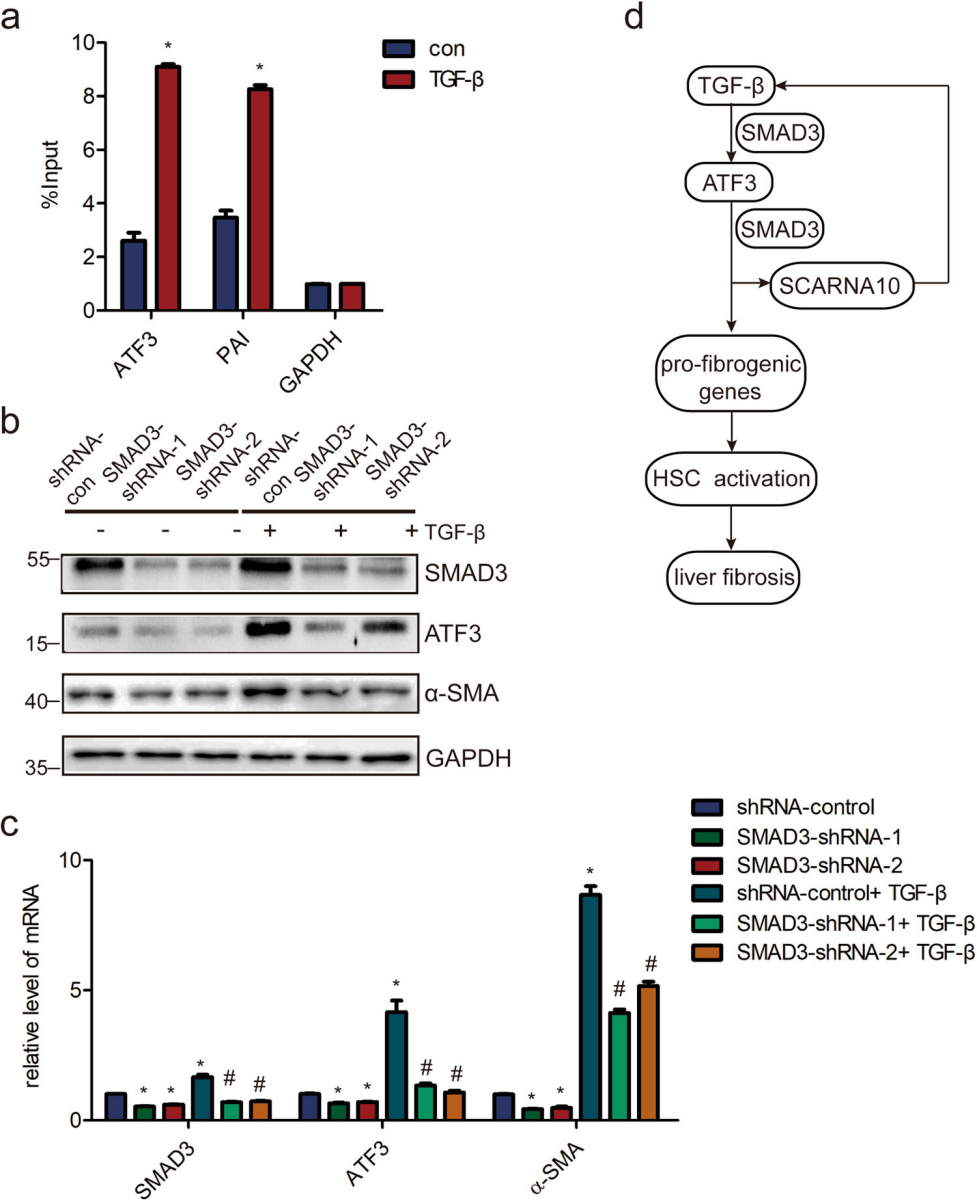
ATF3 is over-expressed in a TGF-β/SMAD3-dependent manner. **a** LX-2 cells were treated with TGF-β and ChIP analyses were performed on ATF3 promoter regions, using anti-SMAD3 antibody. Enrichment was shown relative to input; *p < 0.05. **b**, **c** LX-2 cells were infected with lentivirus-mediated shSMAD3-1/2 for 72 h and further treated with 10 ng/ml TGF-β for additional 24 h. The level of SMAD3, ATF3, and α-SMA were detected by western blot and qRT-PCR. **d** Schematic representation of the TGFβ/ATF3/lnc-SCARNA10 pathway and its function in the progression of liver fibrosis. The data were expressed as the mean ± SEM for at least triplicate experiments. GAPDH was used as an internal control; */#*p* < 0.05, *p < 0.05 for vs shRNA control, # *p* < 0.05 for vs shRNA control + TGF-β.

Since TGF-β signaling was activated by SCARNA10, and previous results showed that ATF3 was increased in LX-2 cells and primary HSCs when treated with TGF-β, we next investigated the mechanism of ATF3 expression induced by TGF-β signaling. Notably, the data showed that the level of SMAD3 was significantly increased binding with ATF3 promoter in response to TGF-β by ChIP assay (Fig. 7a). Most importantly, knockdown of SMAD3 abrogated the stimulatory effects of TGF-β on ATF3 in LX-2 cells (Fig. 7b, c), demonstrating that TGF-β induced ATF3 expression via the canonical TGF-β/SMAD3 pathway. Taken together, these results demonstrate that TGF-β/ATF3/lnc-SCARNA10 form an axis in liver fibrosis.

## Discussion

Emerging as a key regulator of the cell stress response, ATF3 has been shown to involve in multiple physiological and pathological processes by activating signaling or regulating the expression of target genes required for mounting appropriate cellular response^38–40^. Although many molecules have been elucidated in the liver fibrosis-regulated events^41^, neither the functions of ATF3 nor mechanisms by which ATF3 was induced in liver fibrosis has been extensively explored. Here, we have identified that transcription factor ATF3 as one of the upregulated mRNAs in both mice and human fibrotic livers, in activated HSCs and injured HCs. Furthermore, reducing ATF3 expression ameliorated CCl_4_-induced liver fibrosis by inhibiting HSCs activation in vivo and the findings were confirmed in vitro. In addition, we have revealed that ATF3 interacted with SMAD3 to promote the transcription of pro-fibrogenic genes and TGF-β/ATF3/lnc-SCARNA10 formed a positive feedback loop, which contributed to liver fibrosis (Fig. 7d). Our data indicated that ATF3 was crucial for HSCs activation and liver fibrosis, which may provide a potential therapeutic target against liver fibrosis.

Reports concerning the expression and function of ATF3 in liver diseases remain scarce so far. Kim has found that ATF3 was highly expressed in the livers of ZDF rats and in human participants with NAFLD and/or T2D^24^. The results showed that insulin resistance and hepatic steatosis were associated with increased ATF3 expression and decreased fatty acid oxidation via mitochondrial dysfunction, which were attenuated by in vivo ATF3 silencing, suggesting that ATF3 may be a useful biomarker for predicting the progression of NAFLD and the development of T2D, and a potential central strategy for preventing these diseases. In the current study, we demonstrated that ATF3 was significantly increased in mice and human fibrotic livers. Various studies have shown that physiological role of ATF3 is beneficial or detrimental in the development of diseases and cell dysfunction. Our data revealed the potential role of ATF3 that promoted HSCs activation and liver fibrosis by increasing the expression of pro-fibrotic genes. However, the expression of Bax, Mcp1, Bcl2, and Pcna was not significantly decreased in CCl_4_ group when ATF3 was knocked down. Moreover, although ATF3 was upregulated in the injured HCs, the expression of apoptosis and proliferation genes showed no significant difference in the primary HCs isolated from ATF3-shRNA+CCl_4_ group compared with that of CCl_4_ group, in accordance with the in vitro results, suggesting that ATF3 may be not involved in the apoptosis and proliferation of HCs. Thus, enhanced level of ATF3 may be a consequence in injured HCs. All these data indicate that ATF3 aggravates liver fibrosis through promoting the expression of the pro-fibrotic genes and activation of HSCs, rather than regulating the apoptosis and proliferation in HCs.

Studies have reported that ATF3 controls JNK activity through transcriptional regulation of Raw expression, thereby restraining epithelial cell death, a function that is essential for normal gut homeostasis and optimal survival following infection^42^. Surprisingly, the key role of a single transcription factor, ATF3, in determining the extent of liver fibrosis impressed us, because liver fibrosis is a complex disease associated with multiple molecules and signalings. In line with the previous study, our data showed that upon activation, the nuclear localization of ATF3 was increased and we found an ATF3-binding peak in the promoter of target genes, which enhanced the transcription of pro-fibrotic genes. Interestingly, it was newly found that ATF3 promotes the transcription of lnc-SCARNA10, which showed a positive mutual regulation with ATF3. Since the evidence has been illustrated that lnc-SCARNA10 was involved in promoting liver fibrosis, the transcriptional promotion of lnc-SCARNA10 by ATF3 provided a novel mechanism. Previous study has shown that lnc-SCARNA10 promotes the expression of target genes by physically binding with PRC2, thus releasing the *trans*-repression, which may explain the mechanism that lnc-SCARNA10 *trans*-activates ATF3^35^. On the other hand, given the evidence that lnc-SCARNA10 promotes the expression of TGF-β components, thus activating TGF-β signaling, which could subsequently upregulates the level of ATF3, we proposed that TGF-β/ATF3/lnc-SCARNA10 form a positive feedback loop that activates HSCs, contributing to liver fibrosis.

In summary, we have provided, for the first time, evidence that overexpression of ATF3 results in liver fibrosis. Mechanistically, we demonstrated that ATF3 binds the promoter of target genes, in a SMAD3-dependent manner, to *trans*-activate the pro-fibrotic genes and lnc-SCARNA10, which subsequently forms a positive feedback loop with TGF-β and ATF3. Our data suggest the TGF-β/ATF3/lnc-SCARNA10 axis contributes to liver fibrosis by activating HSCs, which broaden the regulatory network of ATF3 and deepen our understanding on the molecular mechanism of liver fibrosis, providing that the modulation of ATF3 in activated HSCs may represent a novel therapeutic approach against liver fibrosis.

## Materials and methods

### Cell culture

The human HSCs line LX-2 (purchased from Merck Millipore, Beijing, China) and HEK-293T (purchased from Chinese Academy of Sciences cell bank, Shanghai, China), were cultured in DMEM (Invitrogen, Camarillo, CA) supplemented with 10% FBS (Gibco, Gaithersburg, MD, USA), penicillin (100 U/ml), and streptomycin (100 μg/ml). The non-tumorigenic mouse hepatocyte cell line AML12 (purchased from Chinese Academy of Sciences cell bank, Shanghai, China) was maintained in DMEM supplemented with 10% FBS, 1× insulin-transferrin-sodium selenite media supplement (Sigma-Aldrich), dexamethasone (40 ng/ml), penicillin (100 U/ml), and streptomycin (100 μg/ml). Cell lines were authenticated by STR profiling, tested negative for mycoplasma. Primary mouse HSCs and HCs were isolated by pronase/collagenase perfusion digestion followed by subsequent density gradient centrifugation, as previously described^43^.

### Construction of plasmids

Oligos encoding shRNA specific for ATF3 and the negative control shRNA were subcloned into the lentiviral shuttle producing the lenti-shATF3 and lenti-NC. The full-length ATF3 cDNA was sequentially amplified by PCR, and ligated into the lentiviral shuttle to generate the LV-ATF3 (overexpression plasmid) and the empty plasmid as the LV-Control. HEK-293T cells were used to produce lentivirus with the knockdown or overexpression plasmids and the packaging plasmids. Recombinant lentiviruses used in vivo were concentrated 100-fold by ultracentrifugation (2 h at 120,000 × *g*). Mice were injected with the virus-containing pellet that was dissolved in PBS within 48 h. Specific siRNAs were transfected with primary HSCs. The primer sets used are shown in Table 1.

**Table 1:** Cloning primers for ATF3, and shRNA and siRNA sequences.

Name	Sequence 5′–3′
Cloning primers for ATF3	
ATF3 (human) 5′ BamHI F	CGCGGATCCATGATGCTTCAACACCCAG
ATF3 (human) 5′ BamHI R	CGCGGATCCTTAGCTCTGCAATGTTCCTTC
ATF3 (mouse) 5′ BamHI F	CGCGGATCCATGATGCTTCAACATCCAGG
ATF3 (mouse) 5′ BamHI R	CGCGGATCCTTAGCTCTGCAATGTTCCTTC
shRNA and siRNA sequences	
Mouse shATF3-1 forward	GATCCCCGGAACCTCTTTATCCAACATTCAAGAGATGTTGGATAAAGAGGTTCCTTTTTA
Mouse shATF3-1 reverse	AGCTTAAAAAGGAACCTCTTTATCCAACATCTCTTGAATGTTGGATAAAGAGGTTCCGGG
Mouse shATF3-2 forward	GATCCCCGCATCCTTTGTCTCACCAATTCAAGAGATTGGTGAGACAAAGGATGCTTTTTA
Mouse shATF3-2 reverse	AGCTTAAAAAGCATCCTTTGTCTCACCAATCTCTTGAATTGGTGAGACAAAGGATGCGGG
Human shATF3-1 forward	GATCCCCGAAGAAGGAGAAGACGGAGTTCAAGAGACTCCGTCTTCTCCTTCTTCTTTTTA
Human shATF3-1 reverse	AGCTTAAAAAGAAGAAGGAGAAGACGGAGTCTCTTGAACTCCGTCTTCTCCTTCTTCGGG
Human shATF3-2 forward	GATCCCCGAGGCGACGAGAAAGAAATTTCAAGAGAATTTCTTTCTCGTCGCCTCTTTTTA
Human shATF3-2 reverse	AGCTTAAAAAGAGGCGACGAGAAAGAAATTCTCTTGAAATTTCTTTCTCGTCGCCTCGGG
Negative control forward	GATCCCCGTTCTCCGAACGTGTCACGTTCAAGAGACGTGACACGTTCGGAGAACTTTTTA
Negative control reverse	AGCTTAAAAAGTTCTCCGAACGTGTCACGTCTCTTGAACGTGACACGTTCGGAGAACGGG
Mouse siATF3-1(516) forward	GCGGCGAGAAAGAAAUAAATT
Mouse siATF3-1(516) reverse	UUUAUUUCUUUCUCGCCGCTT
Mouse siATF3-2(356) forward	CACCCUUUGUCAAGGAAGATT
Mouse siATF3-2(356) forward	UCUUCCUUGACAAAGGGUGTT

### Animals’ in vivo study

Animal protocols were approved by Tianjin Medical University Animal Care and Use Committee. The methods were carried out in accordance with the approved guidelines. All Balb/c male mice aged at 8 weeks obtained from Institute of Laboratory Animal Sciences, CAMS and PUMC (Beijing, China), weighting ~20 g. The liver fibrosis mice model was established by two injections of CCl_4_ (Sigma-Aldrich, St. Louis, MO, USA) per week for 2–8 weeks. Sixty Balb/c mice were randomly divided into six groups: mice were treated with oil in combination with injection of lenti-NC (NC, *n* = 10), oil in combination with injection of lenti-shATF3-1 (shATF3-1, *n* = 10) and lenti-shATF3-2 (shATF3-2, *n* = 10), CCl_4_ in combination with injection of lenti-NC (NC + CCl_4_, *n* = 10), and CCl_4_ in combination with injection of lenti-shATF3-1 (shATF3-1 + CCl_4_, *n* = 10) and lenti-shATF3-2 (shATF3-2 + CCl_4_, *n* = 10). Mice were injected with the lentivirus via the tail vein 2 weeks after the first injection of CCl_4_ (1 × 10^9^ p.f.u. per mouse), then mice were administered 5% CCl_4_ (v/v) dissolved in olive oil (0.2 ml kg^−1^ bodyweight) twice per week for additional 6 weeks via intraperitoneal injection (for the NC + CCl_4_ group, the shATF3-1 + CCl_4_ and shATF3-2 + CCl_4_ group, separately). An equivalent volume of olive oil were injected with the animals in NC, shATF3-1, and shATF3-2 group. All of mice were sacrificed 48 h after the last dose under sodium pentobarbital anesthesia for subsequent experiments. Researchers have always been aware of the grouping of animal experiments.

### Nuclear–cytoplasmic fractionation

Cytoplasmic and nuclear RNA and protein isolation were performed with PARIS™ Kit (Invitrogen, Grand Island, NY, USA), following the manufacturer’s instruction and were performed essentially, as described previously^44^.

### Chromatin immunoprecipitation

ChIP assays were performed essentially as described previously^35^. Briefly, LX-2 cells were infected with shRNA control or lenti-shATF3-1 for 72 h, or treated with or without TGF-β for 24 h, and seeded in cell cultures. After the procedure of cross-link, fixation the cells were harvested in 670 μl SDS buffer containing protease inhibitors (PMSF). Samples were centrifuged and then sheared by sonication with a 5 s/15 s cycle at power setting 30% for 40 times. The supernatant was obtained after centrifuged at 20,000 × *g* for 30 min, and transferred to new tubes and quantified the protein content from each sample by the BCATM Protein Assay Kit using BSA as standard. Next, each single IP was performed in 1 ml volume (samples diluted to a desired concentration and remove 10 μl (1%) of lysate as total control). Lysates were incubated with primary antibodies (5 μg ATF3 (rabbit monoclonal, CST, #8685), 5 μg SMAD2/3, or 5 μg IgG) overnight and subsequently 50 μl beads 4 h at 4 °C. Next, immunoprecipitated complexes were incubated with solution (120 μl of 1% SDS, 0.1 M NaHCO_3_) overnight at 65 °C. Finally, DNA purified with PCR purification kit (Qiagen) were used as templates for PCR reactions. Primers used for PCR in ChIP experiments are described in Table 2.

**Table 2:** Primers for human ChIP qRT-PCR.

Locus	Forward 5′–3′	Reverse 5′–3′
ATF3 (−375 to 176)	TCCCCGTTCCTCCCTATGAC	AGTGCATGTCTGGCCTTCTG
ATF3 (−373 to 171)	CCCGTTCCTCCCTATGACTG	ATGTCTGGCCTTCTGTAGCC
ATF3 (−589 to 152)	GTTCATTCCGCTGTAGCGTC	GGCTTGACACTGATCTGGGG
COL1α1 (−709 to 525)	CCTAGGGTTTGGAGGAAGGC	GTCTTCTGGTGTGGCTAGGG
COL1α1 (−1263 to 1086)	GCATAGAGCAATGACCGGGA	GCCCCTTCTCCAGTTGTACC
COL1α1 (−1846 to 1619)	CTGCCACATGGTCGGGATAA	TGGTTTTGTGCAACGAAGGC
COL3α1 (−186 to 47)	TGCATACAAACTCCAGATGTGC	CCTCACTTTCCAGCCCCTTT
COL3α1 (−698 to 478)	TGTCTTTCCCAGGCAGCATA	AGAAATGCCACCGTATGCCC
COL3α1 (−1819 to 1693)	CCCAAGCAGTGACTCTCCAA	TCAGTCACAAGGACACAAACCA
α-SMA (−245 to 65)	ACTCAGGCAGCGACTTACAG	AAGCAGTGGTTAAGCCGGAG
α-SMA (−1105 to 975)	CAGCCTCTGGTAAAGGTGCTA	ACTGCAATGTTGGCTGCTTTG
α-SMA (−1632 to 1409)	CACCCATCTATGTCCAGCCC	AAATTGGTTTTGCGCTCACGA
MMP2 (−270 to 161)	GGCCCCTGACTGCTCTATTT	TCCCAGGTTGCTTCCTTACC
MMP2 (−975 to 754)	TGTTCCCTAAAACATTCCCC	GTCTCTGAGGAATGTCTTCT
MMP2 (−1821 to 1573)	TGAAGGGAGTCACATACAAGGC	GGCCTGTGGGCTAAATCCA
TIMP1 (−496 to 335)	ATTTGAGACCCTGGCTTTGG	GCAGCAGTGGAGGGAGATAA
TIMP1 (−961 to 754)	ATCAGAACCCCAGGGAAGGT	TGGTGCGGGTGAATGAATGA
TIMP1 (−1619 to 1426)	CACGCCTGTAATCCCAACAC	CCTCCGGGGTTCAAGAGATT
SCARNA10 (−288 to 70)	CCCAGCTTGCCACCGTATTT	CCCCTGACCCATAAACACCTTT
SCARNA10 (−505 to 70)	GCTTTTCAGGCTGCCTTTCG	CCCCTGACCCATAAACACCT
ATF3-pro	GTTCATTCCGCTGTAGCGTC	GGCTTGACACTGATCTGGGG
PAI	CTCCAACCTCAGCCAGACAA	TCCGATGATACACGGCTGAC
GAPDH (−2813 to 2524)	GGTAGGGAGTTCGAGACCAG	TCAACGCAGTTCAGTTAGGC

### qRT-PCR

RNA was isolated from the tissue and cells by TRIzol reagent (TaKaRa, Dalian, China). First-strand cDNA from an equal amount of the RNA sample was synthesized by first-strand synthesis kit #K1622 according to the manufacturer’s instructions (Thermo Fisher Scientific, Waltham, MA, USA) were performed essentially, as described previously^45^. The experiments were repeated three times. Primers are shown in Table 3.

**Table 3:** qRT-PCR primers for analysis of transcript levels.

Gene symbol	Forward 5′–3′	Reverse 5′–3′
Atf3 (mouse)	CTCTGCCATCGGATGTCCTC	GTTTCGACACTTGGCAGCAG
Gapdh (mouse)	GGCATGGACTGTGGTCATGAG	TGCACCACCAACTGCTTAGC
Col1α1 (mouse)	ATCGGTCATGCTCTCTCCAAACCA	ACTGCAACATGGAGACAGGTCAGA
Col3α1 (mouse)	TGCTCCAGTTAGCCCTGCAA	GGTCCTGCAGGCAACAGTGGTTC
Col4α5 (mouse)	CTCCCTTACCGCCCTTTTCTC	AGGCGAAATGGGTATGATGGG
α-SMA (mouse)	TCGGATACTTCAGCGTCAGGA	GTCCCAGACATCAGGGAGTAA
TIMP1 (mouse)	TCCGTCCACAAACAGTGAGTGTCA	GGTGTGCACAGTGTTTCCCTGTTT
MMP2 (mouse)	GTGTTCTTCGCAGGGAATGAG	GATGCTTCCAAACTTCACGCT
Pcna (mouse)	TTTGAGGCACGCCTGATCC	GGAGACGTGAGACGAGTCCAT
Bax (mouse)	TTGCTGATGGCAACTTCAAC	GATCAGCTCGGGCACTTTAG
Mcp1 (mouse)	GTTAACGCCCCACTCACCTG	GGGCCGGGGTATGTAACTCA
Bcl2 (mouse)	GCTGGGATGCCTTTGTGGAACT	CAGAGACAGCCAGGAGAAATCAAAC
ATF3 (human)	CTAACCTGACGCCCTTTGTC	ACTCTTTCTGCAGGCACTCC
GAPDH (human)	ACCCAGAAGACTGTGGATGG	TTCAGCTCAGGGATGACCTT
COL1α1 (human)	AACCAAGGCTGCAACCTGGA	GGCTGAGTAGGGTACACGCAGG
COL3α1 (human)	AGCCTTGCGTGTTCGATAT	GAAGATGTCCTTGATGTGC
COL4α5 (human)	TTCAGCGTTTCTGACTGAGG	AGAGCATCCAGCCATTCATT
α-SMA (human)	GCCATGTTCTATCGGGTACTTC	CAGGGCTGTTTTCCCATCCAT
TIMP1 (human)	GGGGCTTCACCAAGACCTAC	GGAAGCCCTTTTCAGAGCCT
MMP2 (human)	GTGTTCTTCGCAGGGAATGAG	GATGCTTCCAAACTTCACGCT
MMP9 (human)	CCTTGTGCTCTTCCCTGGAG	GGCCCCAGAGATTTCGACTC
SMAD3 (human)	CCCCAGCACATAATAACTTGG	AGGAGATGGAGCACCAGAAG
SCARNA10 (human)	CCAGGGAGGAATTGTGGTAA	TCTGTGTGTCATCTCTCAGTGG

### Confocal microscopy

Immunofluorescence analysis was performed as described previously. The primary antibodies involved in this study include Col1α1 (1:500, Abcam, ab34710) and ATF3 (rabbit polyclonal, GeneTex, GTX30069). Secondary antibodies conjugated with Alexa Fluor 488 were incubated in PBS away from light (Thermo Fisher Scientific, Alexa Fluor 488) for 1 h and DAPI for 20 min at room temperature. The stained cells were observed with a Zeiss confocal microscope LSM700.

### Western blot and immunoprecipitation analysis

Whole cell lysate was obtained for immunoprecipitation and immunoblotting according to standard procedures. The primary antibodies involved in this study, including ATF3 (rabbit polyclonal, GeneTex, GTX30069 or rabbit monoclonal, CST, #8685), α-SMA (rabbit polyclonal, Abcam, ab5694), collagen1 (rabbit polyclonal, Abcam, ab34710; Millipore, #234167), MMP2 (rabbit monoclonal, Abcam, ab92536), Smad2/3 (rabbit monoclonal, CST 5678), PCNA (rabbit monoclonal, CST, 13110), BAX (rabbit monoclonal, ab32503), Caspase3 (rabbit polyclonal CST, #9662), H3K27 (mouse monoclonal, Abcam, ab6002), rabbit IgG (Millipore, PP64B), goat anti-rabbit/mouse IgG, and GAPDH (1:8000).

### Hydroxyproline assay

Total collagen content was tested by measuring the amount of hydroxyproline in liver tissue using commercially available hydroxyproline detection kits (Nanjing Jian-cheng Corp., Nanjing, China), according to the manufacturer’s instructions.

### Histology and immunohistochemistry

The immunohistochemistry was performed essentially as described previously^44^. The slides were treated with primary antibody α-SMA (1:50, rabbit polyclonal, Abcam, ab5694), collagen1 (1:1000, rabbit polyclonal, Abcam, ab34710), ATF3 (1:200, rabbit polyclonal, GeneTex, GTX30069), and BAX (1:200, rabbit monoclonal, ab32503) overnight at 4 °C. In addition, tissue sections were processed omitting the primary antibody as the negative control. The slides were then incubated with secondary antibody (1:500; horse radish peroxidase-conjugated anti-rabbit IgG) and the reaction products were visualized, using diaminobenzidine and monitored by microscopy. Morphometrical analysis was performed for five random fields in each preparation, and average percentages of fibrotic area are plotted.

### Study population

In total, 25 human fibrotic liver tissues and 7 human healthy liver tissues from patients with hepatic haemangioma were obtained from surgical resections without preoperative treatment at Tianjin Third Central Hospital (Tianjin, China). Hepatic fibrosis was scored according to the METAVIR fibrosis staging system by three hepatopathologists blinded to the study protocol. Total RNA and protein of the liver tissues were obtained for subsequent qRT-PCR and immunoblotting. All subjects were of the same ethnicity. Clinical and pathological characteristics were recorded and summarized in Table 4. The study has been approved by the local Ethical Committee of Tianjin Third Central Hospital (Tianjin, China). Written informed consent was obtained from each patient according to the policies of the committee. The study methodologies were conformed to the standards set by the Declaration of Helsinki.

**Table 4:** Baseline characteristics of patients with liver tissue.

Metavir score	Healthy (*F*0)	Fibrosis (*F*1–*F*4)
Cases (*n*)	7	25
Age (years)^a^	60.3 ± 14.5	50.4 ± 10.3
Male sex (*n* (%))	4 (57)	12 (48)
ALT (U/L)^a^	21.6 ± 11.3	34.3 ± 24.1
AST (U/L)^a^	25.4 ± 11.8	52.5 ± 48.4
Etiology (*n* (%))		
Biliary obstruction	0 (0)	3 (12)
HBV	0 (0)	12 (48)
HCV	0 (0)	1 (4)

*ALT* alanine aminotransferase, *AST* aspartate aminotransferase, *HBV* hepatitis B virus, *HCV* hepatitis C virus.

^a^Mean ± SD.

### Data analysis

The liver fibrosis gene expression GEO dataset was downloaded from the GEO database (GSE80601). Significant analysis of microarray software was used to analyze differentially expressed mRNAs between normal liver tissues and fibrotic liver tissue of mice. The cutoff value for differentially expressed mRNA was set to a >2-fold difference, and the *p* value was < 0.05.

### Statistical analysis

Data were expressed as mean ± SD. All the statistical analyses were performed with the SPSS 13.0 (IBM, Armonk, NY, USA). Statistical analyses were performed using either Student’s *t* test (two-group comparison) or one-way analysis of variance (more than two groups) followed by post hoc comparison, and differences with *p* < 0.05 were considered significantly.

## Supplementary information

supplementary figure legends

supplementary figure 1

supplementary figure 2

supplementary figure 3

supplementary figure 4

supplementary figure 5

supplementary figure 6

supplementary figure 7

supplementary figure 8

supplementary figure 9

supplementary figure 10

## References

[1] Bottcher K, Pinzani M (2017). Pathophysiology of liver fibrosis and the methodological barriers to the development of anti-fibrogenic agents. Adv. Drug Deliv. Rev..

[2] Li Z (2019). MKL1 promotes endothelial-to-mesenchymal transition and liver fibrosis by activating TWIST1 transcription. Cell Death Dis..

[3] Wandrer F (2020). TNF-Receptor-1 inhibition reduces liver steatosis, hepatocellular injury and fibrosis in NAFLD mice. Cell Death Dis..

[4] Jia D (2019). SVIP alleviates CCl4-induced liver fibrosis via activating autophagy and protecting hepatocytes. Cell Death Dis..

[5] Rezvani M (2016). In vivo hepatic reprogramming of myofibroblasts with AAV vectors as a therapeutic strategy for liver fibrosis. Cell Stem Cell.

[6] Ray K (2019). HAstening the development of liver fibrosis. Nat. Rev. Gastroenterol. Hepatol..

[7] Zhang F, Kong D, Lu Y, Zheng S (2013). Peroxisome proliferator-activated receptor-gamma as a therapeutic target for hepatic fibrosis: from bench to bedside. Cell Mol. Life Sci..

[8] Wang X (2020). Roseotoxin B alleviates cholestatic liver fibrosis through inhibiting PDGF-B/PDGFR-beta pathway in hepatic stellate cells. Cell Death Dis..

[9] Tsuchida T, Friedman SL (2017). Mechanisms of hepatic stellate cell activation. Nat. Rev. Gastroenterol. Hepatol..

[10] Wang M (2020). Liver-targeted delivery of TSG-6 by calcium phosphate nanoparticles for the management of liver fibrosis. Theranostics.

[11] Sun WY (2020). beta-arrestin2 deficiency protects against hepatic fibrosis in mice and prevents synthesis of extracellular matrix. Cell Death Dis..

[12] McDaniel K (2017). The let-7/Lin28 axis regulates activation of hepatic stellate cells in alcoholic liver injury. J. Biol. Chem..

[13] Ramachandran P (2019). Resolving the fibrotic niche of human liver cirrhosis at single-cell level. Nature.

[14] Bessone F, Razori MV, Roma MG (2019). Molecular pathways of nonalcoholic fatty liver disease development and progression. Cell Mol. Life Sci..

[15] Cui H (2015). The stress-responsive gene ATF3 regulates the histone acetyltransferase Tip60. Nat. Commun..

[16] Hai T, Hartman MG (2001). The molecular biology and nomenclature of the activating transcription factor/cAMP responsive element binding family of transcription factors: activating transcription factor proteins and homeostasis. Gene.

[17] Gilchrist M (2010). A key role for ATF3 in regulating mast cell survival and mediator release. Blood.

[18] Kim KH, Jeong JY, Surh YJ, Kim KW (2010). Expression of stress-response ATF3 is mediated by Nrf2 in astrocytes. Nucleic Acids Res..

[19] Suganami T (2009). Activating transcription factor 3 constitutes a negative feedback mechanism that attenuates saturated Fatty acid/toll-like receptor 4 signaling and macrophage activation in obese adipose tissue. Circ. Res..

[20] De Nardo D (2013). High-density lipoprotein mediates anti-inflammatory reprogramming of macrophages via the transcriptional regulator ATF3. Nat. Immunol..

[21] Janz M (2006). Classical Hodgkin lymphoma is characterized by high constitutive expression of activating transcription factor 3 (ATF3), which promotes viability of Hodgkin/Reed-Sternberg cells. Blood.

[22] Hartman MG (2004). Role for activating transcription factor 3 in stress-induced -cell apoptosis. Mol. Cell. Biol..

[23] Wu X (2010). Opposing roles for calcineurin and ATF3 in squamous skin cancer. Nature.

[24] Kim JY (2017). Activating transcription factor 3 is a target molecule linking hepatic steatosis to impaired glucose homeostasis. J. Hepatol..

[25] Koh IU (2010). AdipoR2 is transcriptionally regulated by ER stress-inducible ATF3 in HepG2 human hepatocyte cells. FEBS J..

[26] Li Y (2017). Cardiac fibroblast-specific activating transcription factor 3 protects against heart failure by suppressing MAP2K3-p38 signaling. Circulation.

[27] Thien A (2015). TSC1 activates TGF-beta-Smad2/3 signaling in growth arrest and epithelial-to-mesenchymal transition. Dev. Cell.

[28] Tang LY (2017). Transforming growth factor-beta (TGF-beta) directly activates the JAK1-STAT3 axis to induce hepatic fibrosis in coordination with the SMAD pathway. J. Biol. Chem..

[29] Palumbo-Zerr K (2015). Orphan nuclear receptor NR4A1 regulates transforming growth factor-beta signaling and fibrosis. Nat. Med..

[30] Kang Y, Chen CR, Massague J (2003). A self-enabling TGFbeta response coupled to stress signaling: Smad engages stress response factor ATF3 for Id1 repression in epithelial cells. Mol. Cell.

[31] Wu X (2016). CUG-binding protein 1 regulates HSC activation and liver fibrogenesis. Nat. Commun..

[32] Su, J. et al. TGF-beta orchestrates fibrogenic and developmental EMTs via the RAS effector RREB1. *Nature* **577**, 566–571 (2020). 10.1038/s41586-019-1897-5 PMC7450666 31915377

[33] Jiang D, Liang J (2019). A long noncoding RNA links TGF-beta signaling in lung fibrosis. Am. J. Respir. Crit. Care Med..

[34] Wang, P. et al. Long noncoding RNA lnc-TSI inhibits renal fibrogenesis by negatively regulating the TGF-beta/Smad3 pathway. *Sci. Transl Med.* **10**, eaat2039 (2018). 10.1126/scitranslmed.aat2039 30305452

[35] Zhang K (2019). SCARNA10, a nuclear-retained long non-coding RNA, promotes liver fibrosis and serves as a potential biomarker. Theranostics.

[36] Zhang K (2017). The liver-enriched lnc-LFAR1 promotes liver fibrosis by activating TGFbeta and Notch pathways. Nat. Commun..

[37] Wang X (2016). Hepatocyte TAZ/WWTR1 promotes inflammation and fibrosis in nonalcoholic steatohepatitis. Cell Metab..

[38] Bambouskova M (2018). Electrophilic properties of itaconate and derivatives regulate the IkappaBzeta-ATF3 inflammatory axis. Nature.

[39] Boespflug ND (2014). ATF3 is a novel regulator of mouse neutrophil migration. Blood.

[40] Hoetzenecker W (2011). ROS-induced ATF3 causes susceptibility to secondary infections during sepsis-associated immunosuppression. Nat. Med..

[41] Pan X-y (2019). Methylation of RCAN1.4 mediated by DNMT1 and DNMT3b enhances hepatic stellate cell activation and liver fibrogenesis through Calcineurin/NFAT3 signaling. Theranostics.

[42] Zhou J, Edgar BA, Boutros M (2017). ATF3 acts as a rheostat to control JNK signalling during intestinal regeneration. Nat. Commun..

[43] Zhang K (2020). Silencing lncRNA Lfar1 alleviates the classical activation and pyoptosis of macrophage in hepatic fibrosis. Cell Death Dis..

[44] Zhang K (2019). The hepatocyte-specifically expressed lnc-HSER alleviates hepatic fibrosis by inhibiting hepatocyte apoptosis and epithelial-mesenchymal transition. Theranostics.

[45] Shi Z (2017). The circular RNA ciRS-7 promotes APP and BACE1 degradation in an NF-kappaB-dependent manner. FEBS J..

