# Supplementary material for: Transcriptional factor ATF3 promotes liver fibrosis via activating hepatic stellate cells
Source: Cell Death Dis. 2020 Dec 14;11(12):1066. doi: 10.1038/s41419-020-03271-6 (PMC7734065; doi:10.1038/s41419-020-03271-6)
Supplement: Supplementary file 1 — supplementary figure legends [file 41419_2020_3271_MOESM1_ESM.docx]

**Supplementary Figure legends**

**Supplementary Figure 1. ATF3 is up-regulated in the progression of liver fibrosis and in the activation of HSCs. a** The differentially expressed genes was showed by the volcano plot according to the microarray data (GSE80601)，the red plot are up-regulated genes and the green plot are down-regulated genes with statistical significance (2.0-fold up and down and p<0.05). **b** The fold change of ATF3 was showed according to the microarray data**. c, d** The expression of ATF3 and α-SMA was measured by qRT-PCR analysis and immunoblot in liver tissues from mice treated with CCl_4_ for 0, 2, 4, 6, 8 or 12 weeks. **e, f** Primary HSCs were isolated from liver tissues of Balb/c mice treated for mice treated with CCl_4_ for 0, 2, 4, 6, 8 or 12 weeks, the expression of ATF3 and α-SMA was measured by qRT-PCR analysis and immunoblot. The data were expressed as the mean ± SEM for at least triplicate experiments. GAPDH was used as an internal control. **p*<0.05.

**Supplementary Figure 2. ATF3 is up-regulated in the injured HCs. a, b** The expression of ATF3 was measured in primary HCs isolated from normal or fibrotic livers by RT-PCR and western blot. **c, d** The expression of ATF3 was measured in primary HCs treated with or without TGF-β by RT-PCR and western blot. **e, f** The expression of ATF3 was measured in AML12 cells treated with or without TGF-β by RT-PCR and western blot. The data were expressed as the mean ± SEM for at least triplicate experiments. GAPDH was used as an internal control. **p*<0.05.

**Supplementary Figure 3. Reducing ATF3 expression inhibits CCl_4_-induced liver fibrosis.** ATF3 expression was determined in the six groups of mice (related to Fig.3) and liver fibrosis was evaluated by IHC for COL1 respectively.

**Supplementary Figure 4. Reducing ATF3 expression inhibits HSCs activation in CCl_4_-induced liver fibrosis and in primary HSCs *in vitro*.** Mice were treated with oil in combination with injection of lenti-NC (Negative Control, n=10), or CCl_4_ in combination with injection of lenti-NC (NC+CCl_4_, n=10), or oil in combination with injection of lenti-ATF3-shRNA1/2 (ATF3-shRNA-1/2, n=10), or CCl_4_ in combination with injection of lenti-ATF3-shRNA1/2 (ATF3-shRNA-1/2+CCl_4_, n=10). **a, b** The mRNA and protein level of ATF3 and pro-fibrogenic genes were measured by qRT-PCR analysis and western blot in the primary HSCs, which were isolated from mice in each group. **c, d** Primary HSCs cells were infected with specific siRNAs of ATF3 for 72 h. **c** The expression of *α-SMA, COL1α1, COL3α1, COL4α5, MMP2, MMP9* and *TIMP1* was detected by qRT-PCR. **p*<0.05. **d** The protein level of α-SMA, COL1 and MMP2 was detected by western blot. GAPDH was used as an internal control. The data were expressed as the mean ± SEM for at least triplicate experiments, GAPDH was used as an internal control. */#*p*<0.05. **p*<0.05 for vs NC or control. #*p*<0.05 for vs NC + CCl_4_.

**Supplementary Figure 5. ATF3 is not involved in the function of HCs. a** The expression of BAX was evaluated in the mice liver of each group by IHC. **b** The mRNA level of *Bax, Mcp1, Bcl2* and *Pcna* was assessed by qRT-PCR analysis in liver tissues. **c** The protein level of BAX, PCNA and Caspase 3 was measured by western blot in liver tissues. **d** The mRNA level of *ATF3, Bax, Mcp1, Bcl2* and *Pcna* was measured by qRT-PCR analysis in the primary HCs isolated from mice in each group. The data were expressed as the mean ± SEM for at least triplicate experiments. GAPDH was used as an internal control. **p*<0.05.

**Supplementary Figure 6. ATF3 is not involved in the apoptosis or proliferation of HCs. a** The mRNA level of *Atf3,* *Bax, Mcp1, Bcl2* and *Pcna* was assessed by qRT-PCR analysis in ATF3-silenced primary HCs. **b** The protein level of BAX, PCNA was measured by western blot in ATF3-silenced primary HCs. **c** The mRNA level of *Atf3,* *Bax, Mcp1, Bcl2* and *Pcna* was assessed by qRT-PCR analysis in ATF3 over-expressed primary HCs. **d** The protein level of BAX, PCNA was measured by western blot in ATF3 over-expressed primary HCs. The data were expressed as the mean ± SEM for at least triplicate experiments. GAPDH was used as an internal control. **p*<0.05.

**Supplementary Figure 7. ATF3 is not involved in the apoptosis or proliferation of AML12 cells. a** The mRNA level of *Atf3,* *Bax, Mcp1, Bcl2* and *Pcna* was assessed by qRT-PCR analysis in ATF3-silenced AML12 cells. **b** The protein level of BAX, PCNA was measured by western blot in ATF3-silenced AML12 cells. **c** The mRNA level of *Atf3,* *Bax, Mcp1, Bcl2* and *Pcna* was assessed by qRT-PCR analysis in ATF3 over-expressed AML12 cells. **d** The protein level of BAX, PCNA was measured by western blot in ATF3 over-expressed AML12 cells. The data were expressed as the mean ± SEM for at least triplicate experiments. GAPDH was used as an internal control. **p*<0.05.

**Supplementary Figure 8. ATF3 promotes the expression of pro-fibrogenic genes in LX-2 cells.** LX-2 cells were infected with lenti-ATF3 shRNA1/2 for 72 h. **a** The expression of *α-SMA, COL1α1, COL3α1, COL4α5, MMP2, MMP9* and *TIMP1* was detected by qRT-PCR. **p*<0.05. **b** The protein level of α-SMA, COL1 and MMP2 was detected by western blot. GAPDH was used as an internal control. **c** The mRNA level of *α-SMA, COL1α1, COL3α1, COL4α5, MMP2, MMP9* and *TIMP1* was detected in LX-2 cells infected with lenti-ATF3 or lenti-control by qRT-PCR. **p* < 0.05. **d** The protein level of α-SMA, COL1 and MMP2 were detected in ATF3 up-regulated LX-2 cells by western blot. GAPDH was used as an internal control. The data were expressed as the mean ± SEM for at least triplicate experiments.

**Supplementary Figure 9. ATF3 up-regulates the pro-fibrotic genes expression induced by TGF-β. a, b** LX-2 cells were infected with lentivirus-mediated ATF3-sh1/2 for 72 h and further treated with 10 ng/ml TGF-β for additional 24 h. The level of pro-fibrotic genes was detected by qRT-PCR and western blot. The data were expressed as the mean ± SEM for at least triplicate experiments. GAPDH was used as an internal control. **p*<0.05.

**Supplementary Figure 10. ATF3 translocates to the nucleus of activated HSCs and form complexes with SMAD3 in primary HSCs. a** Primary HSCs were isolated from NC or CCl_4_ group; primary HSCs were culture activated at day 14; primary HSCs were treated with 10 ng/ml TGF-β for 48 h; the expression and location of ATF3 and COL1 was determined by confocal microscopy. DAPI-stained nuclei blue; scale bar, 20 μm. **b** ATF3 and SMAD3 antibodies were used for co-IP with primary HSCs lysates treated with or without TGF-β. The data were expressed as the mean ± SEM for at least triplicate experiments.
