# Supplementary figures and images for: Transcriptional factor ATF3 promotes liver fibrosis via activating hepatic stellate cells
Source: Cell Death Dis. 2020 Dec 14;11(12):1066. doi: 10.1038/s41419-020-03271-6 (PMC7734065; doi:10.1038/s41419-020-03271-6)

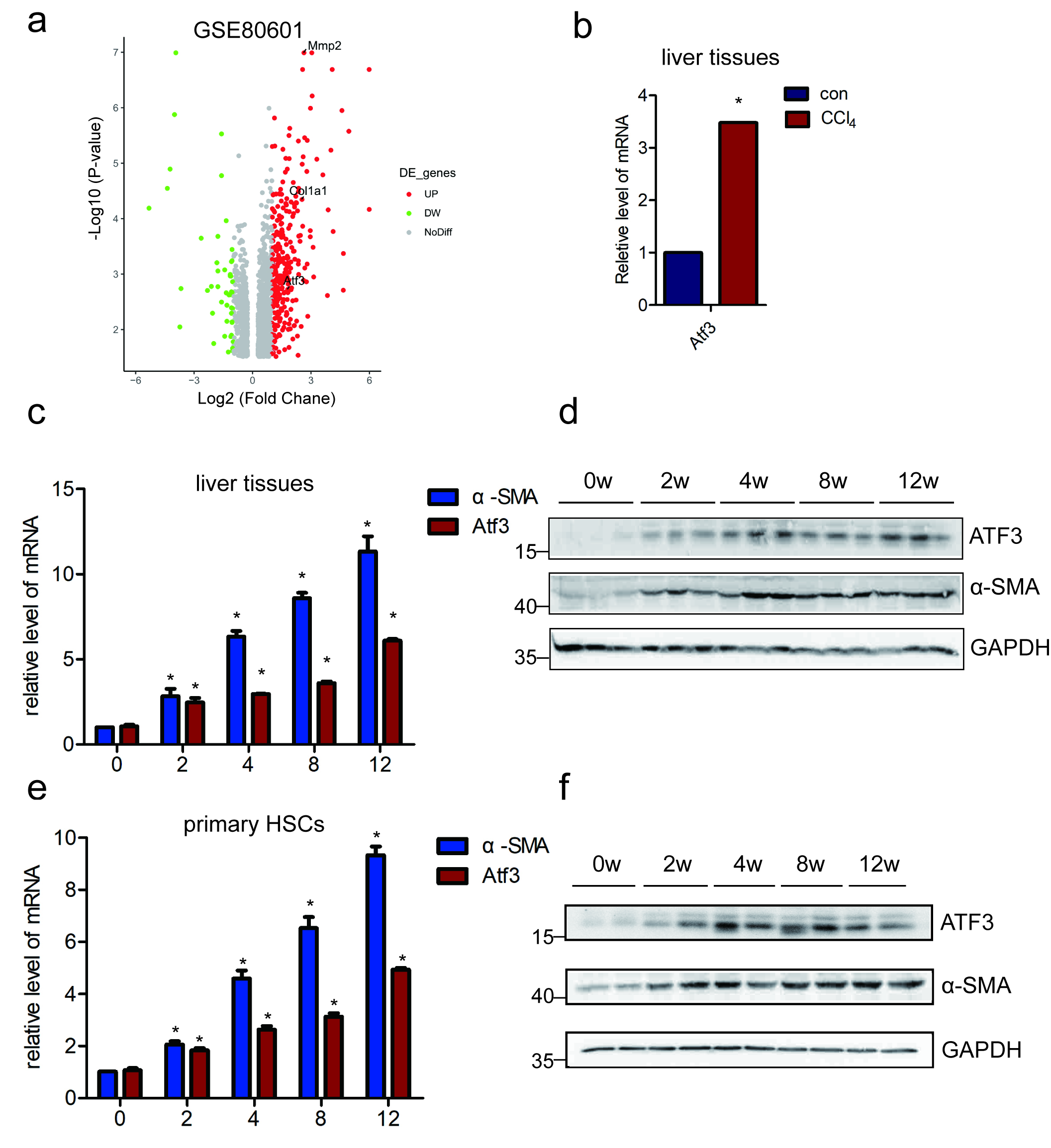

Supplement: Supplementary file 2 — supplementary figure 1 [file 41419_2020_3271_MOESM2_ESM.tif]

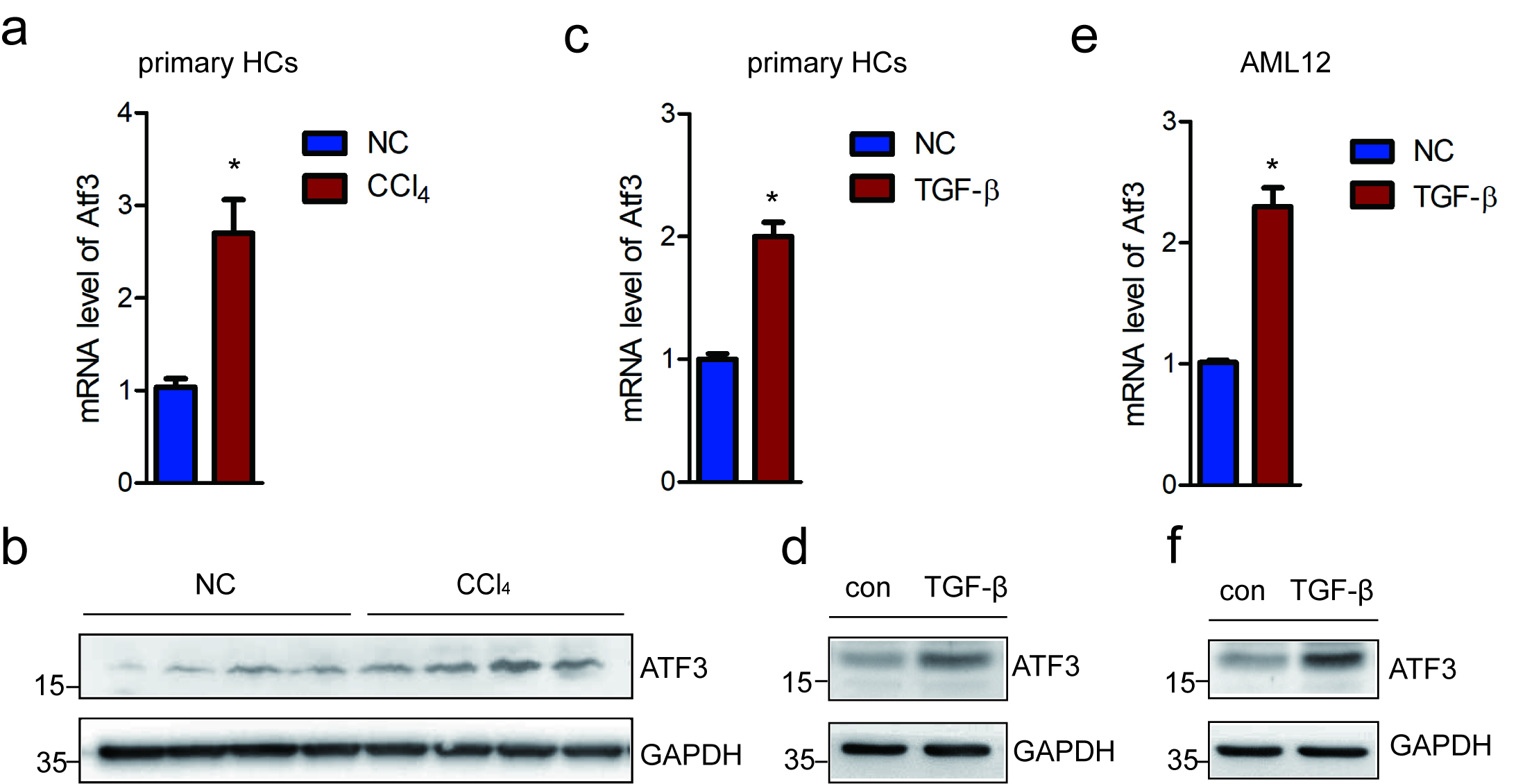

Supplement: Supplementary file 3 — supplementary figure 2 [file 41419_2020_3271_MOESM3_ESM.tif]

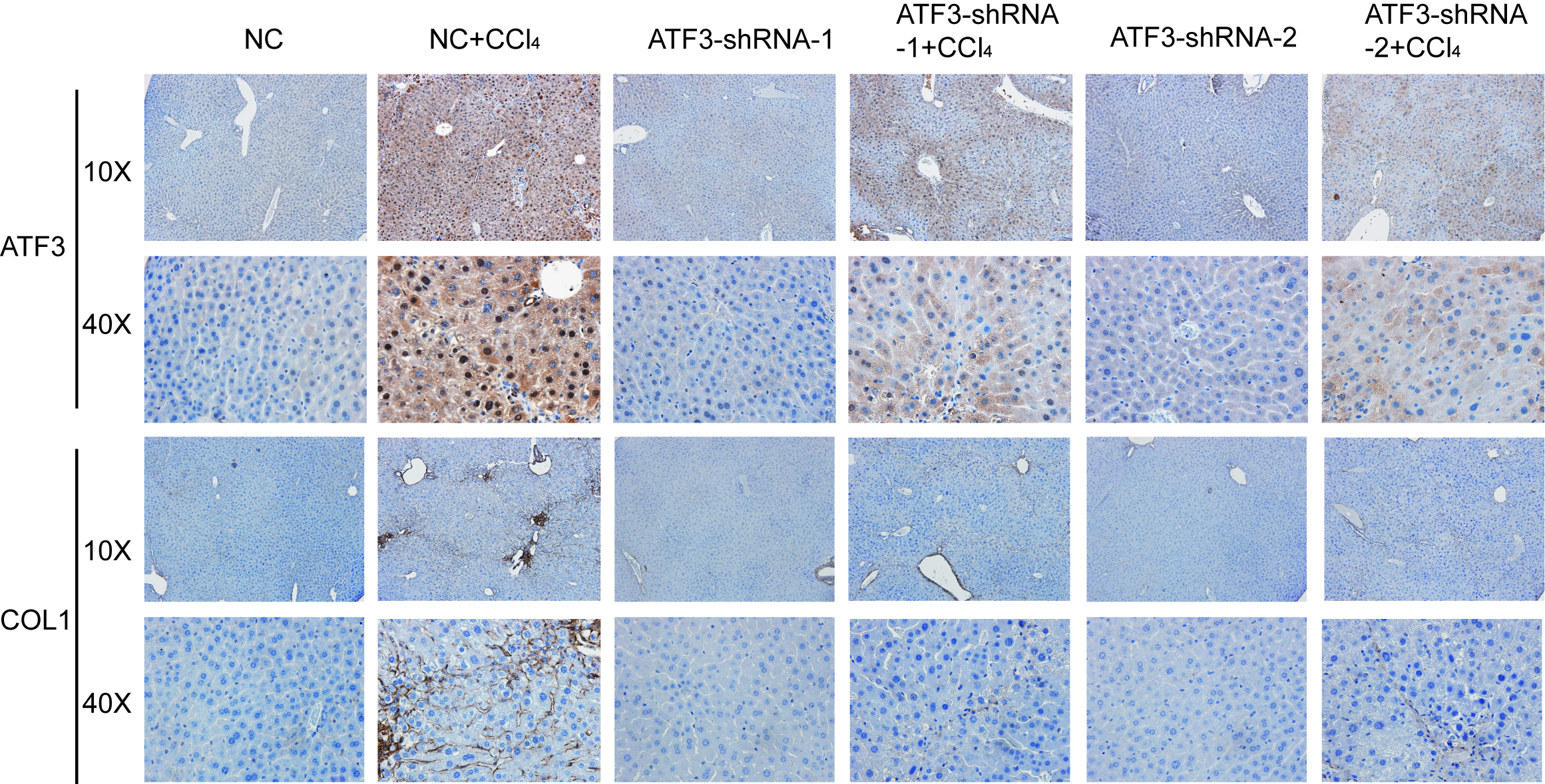

Supplement: Supplementary file 4 — supplementary figure 3 [file 41419_2020_3271_MOESM4_ESM.tif]

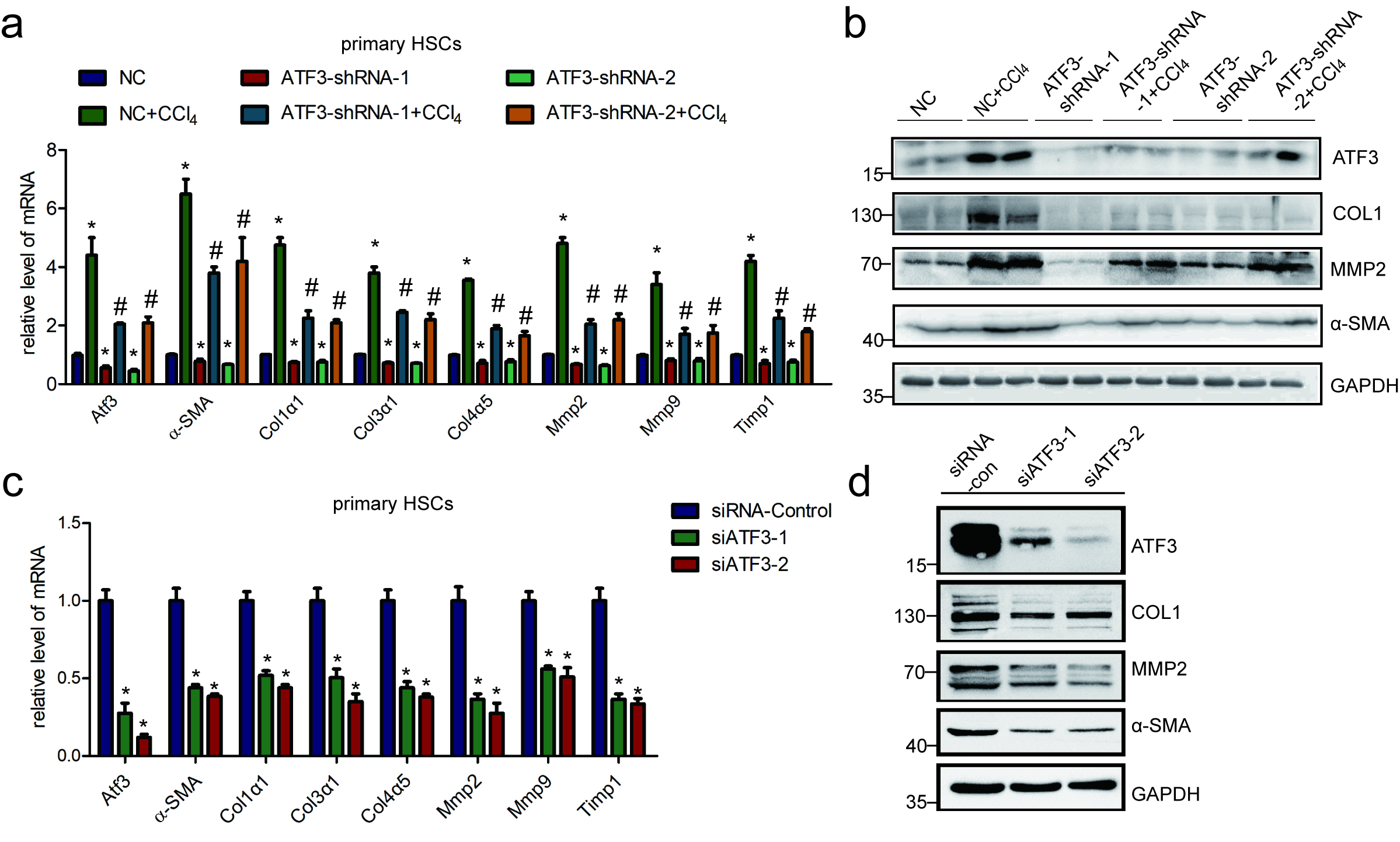

Supplement: Supplementary file 5 — supplementary figure 4 [file 41419_2020_3271_MOESM5_ESM.tif]

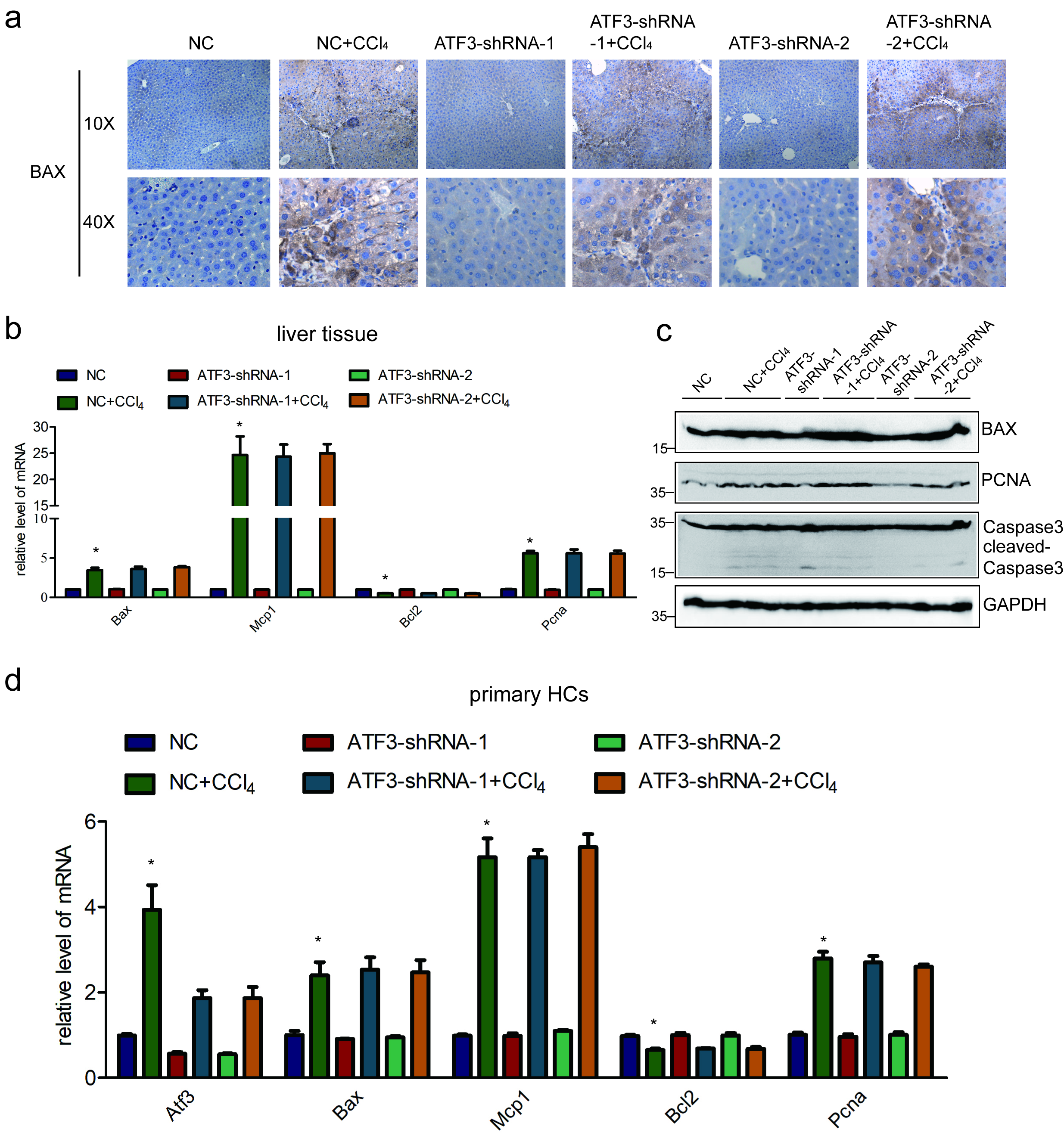

Supplement: Supplementary file 6 — supplementary figure 5 [file 41419_2020_3271_MOESM6_ESM.tif]

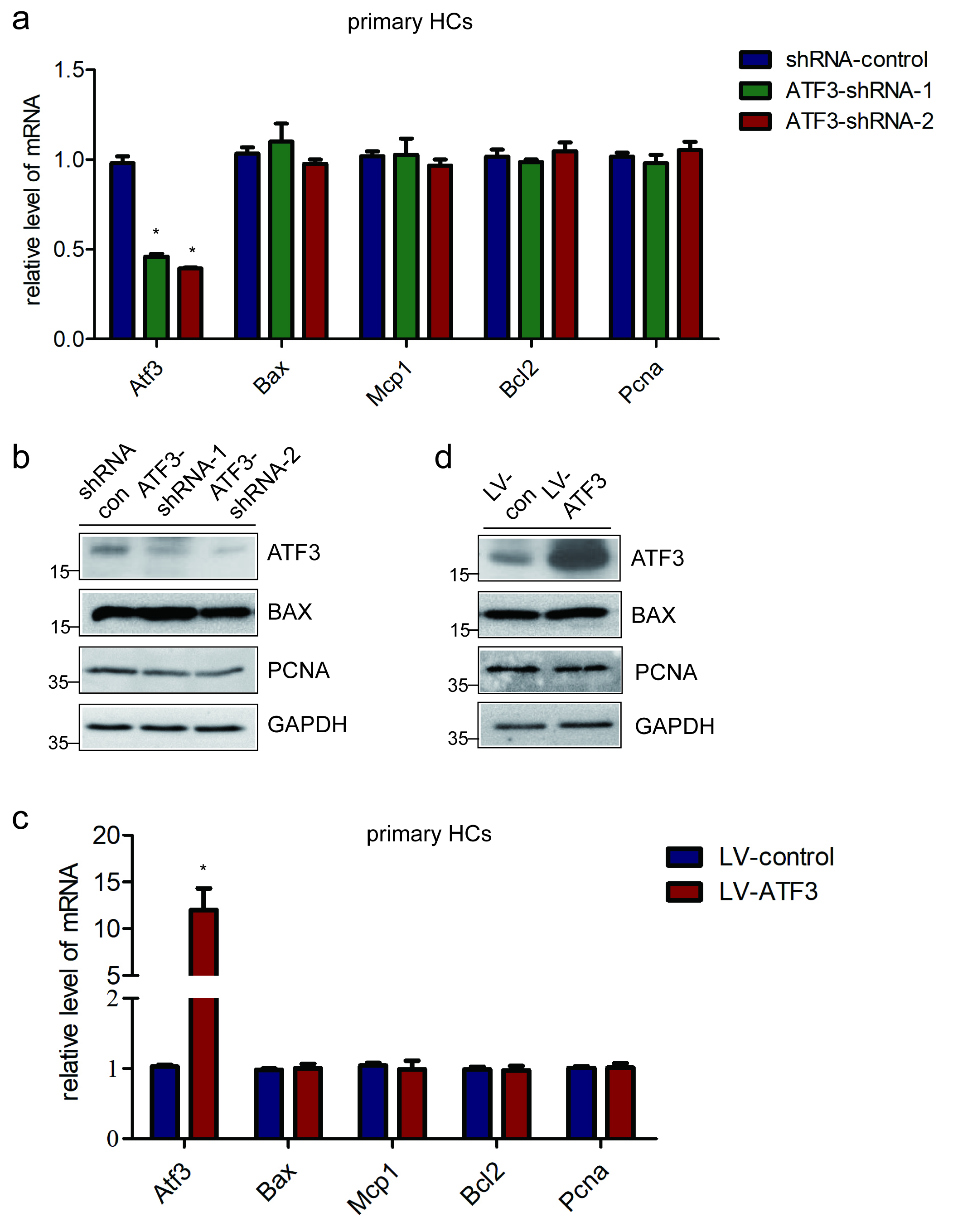

Supplement: Supplementary file 7 — supplementary figure 6 [file 41419_2020_3271_MOESM7_ESM.tif]

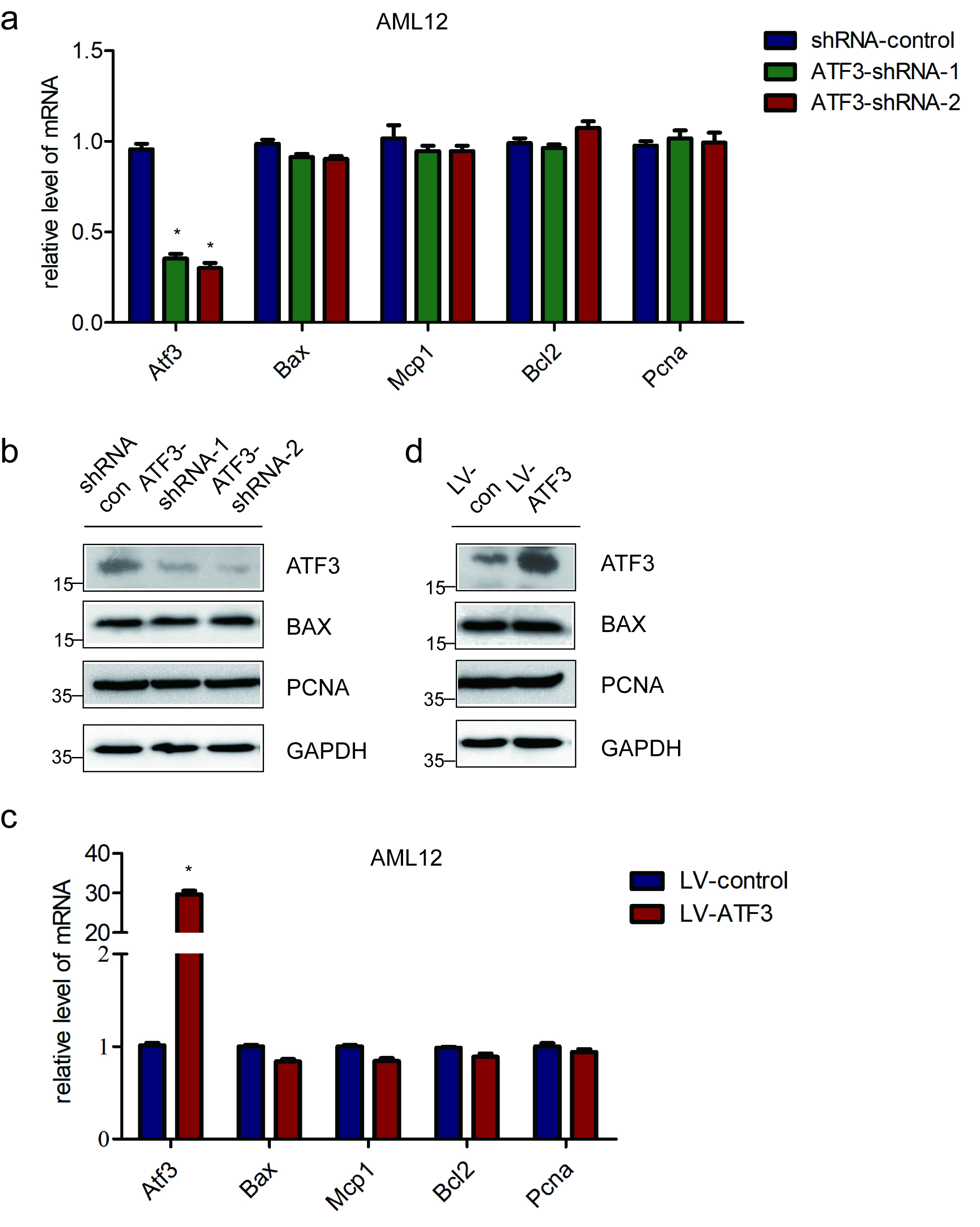

Supplement: Supplementary file 8 — supplementary figure 7 [file 41419_2020_3271_MOESM8_ESM.tif]

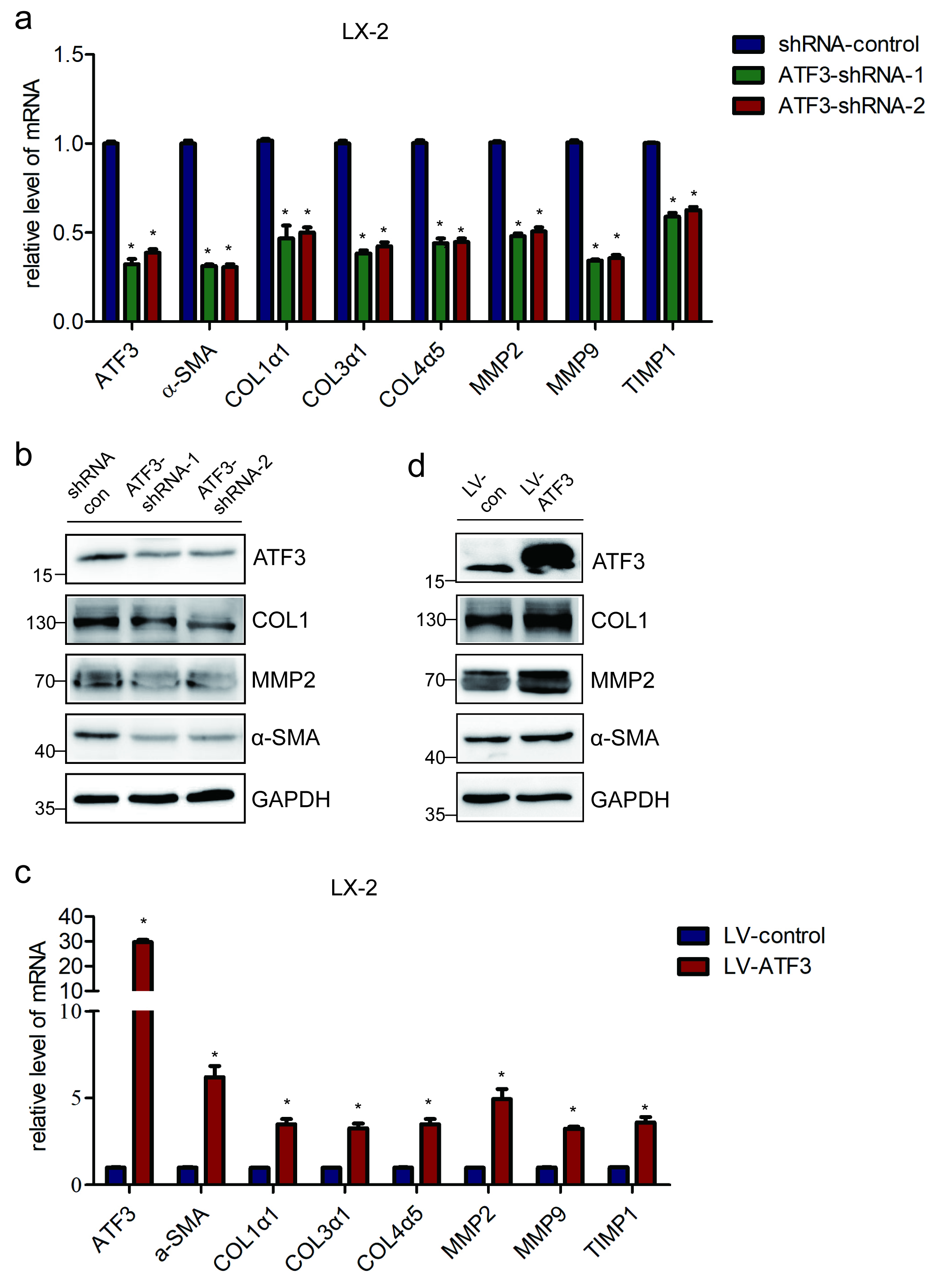

Supplement: Supplementary file 9 — supplementary figure 8 [file 41419_2020_3271_MOESM9_ESM.tif]

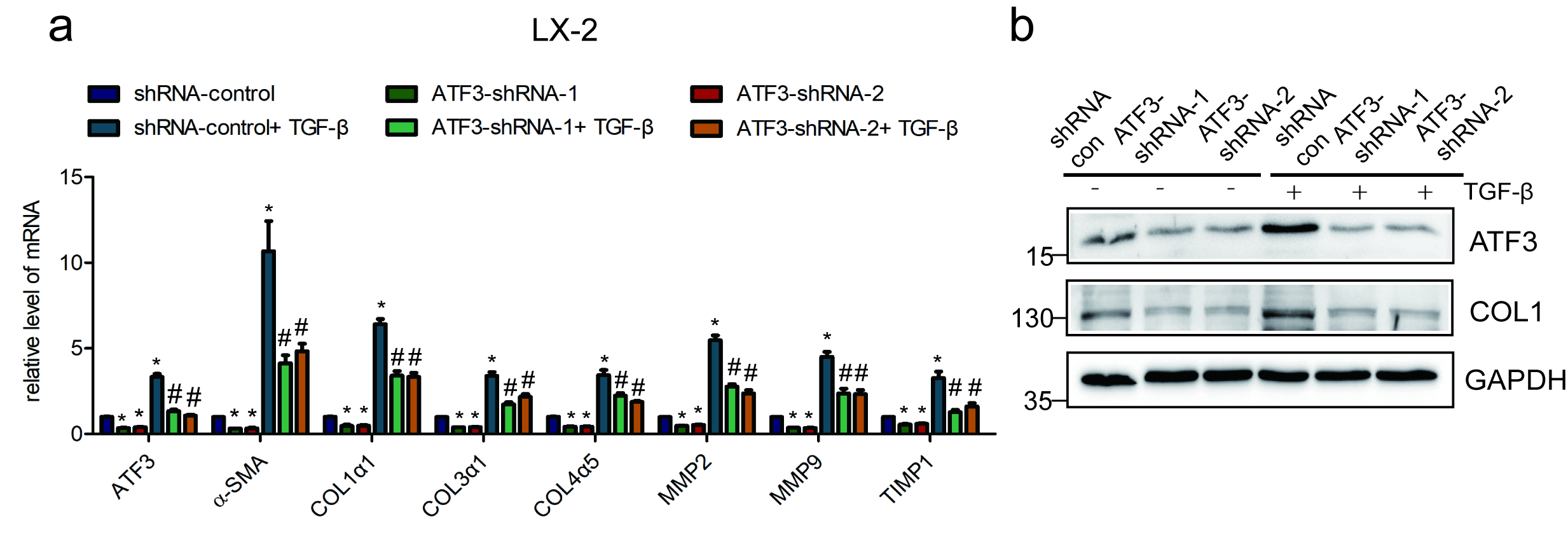

Supplement: Supplementary file 10 — supplementary figure 9 [file 41419_2020_3271_MOESM10_ESM.tif]

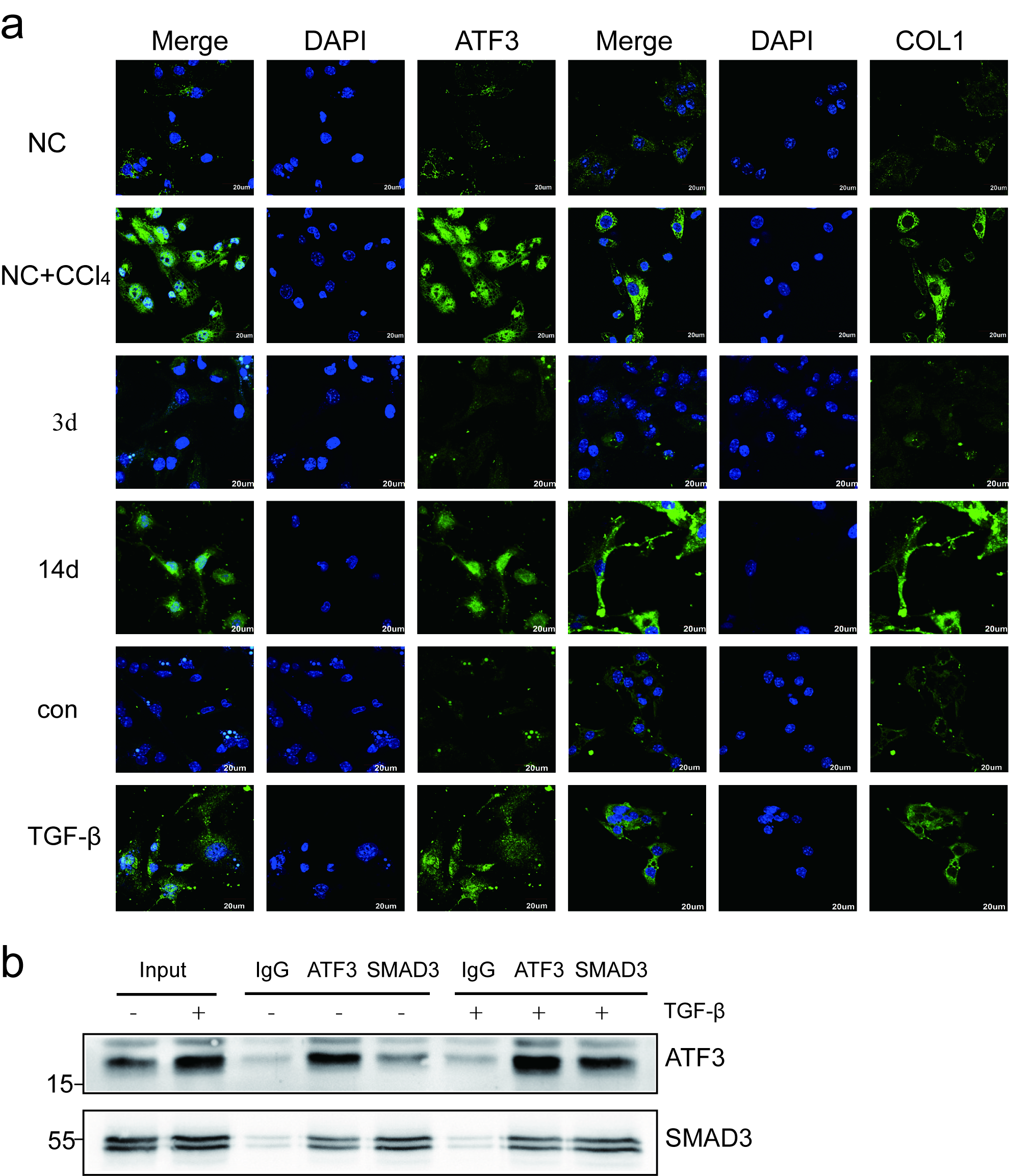

Supplement: Supplementary file 11 — supplementary figure 10 [file 41419_2020_3271_MOESM11_ESM.tif]
